# B Cell-Activating Factor Regulates Different Aspects of B Cell Functionality and Is Produced by a Subset of Splenic B Cells in Teleost Fish

**DOI:** 10.3389/fimmu.2017.00295

**Published:** 2017-03-15

**Authors:** Carolina Tafalla, Lucia González, Rosario Castro, Aitor G. Granja

**Affiliations:** ^1^Laboratory of Fish Immunology and Pathology, Centro de Investigación en Sanidad Animal (CISA-INIA), Madrid, Spain

**Keywords:** teleost, B cells, B cell-activating factor, IgM, cell survival, MHC II

## Abstract

In mammals, B cell functionality is greatly influenced by cytokines released by innate cells, such as macrophages or dendritic cells, upon the early recognition of common pathogen patterns through invariant receptors. B cell-activating factor (BAFF) is one of these innate B cell-helper signals and plays a key role in the survival and differentiation of B cells. Although, evolutionarily, teleost fish constitute the first animal group in which adaptive immunity based on Ig receptors is present, fish still rely greatly on innate responses. In this context, we hypothesized that BAFF would play a key role in the control of B cell responses in fish. Supporting this, our results show that teleost BAFF recapitulates mammalian BAFF stimulating actions on B cells, upregulating the expression of membrane MHC II, improving the survival of fish naïve B cells and antibody-secreting cells, and increasing the secretion of IgM. Surprisingly, we also demonstrate that BAFF is not only produced in fish by myeloid cells but is also produced by a subset of splenic B cells. Thus, if this B cell-produced BAFF proves to be actively regulating this same B cell subset, our findings point to an ancient mechanism to control B cell differentiation and survival in lower vertebrates, which has been silenced in mammals in physiological conditions, but reemerges under pathological conditions, such as B cell lymphomas and autoimmune diseases.

## Introduction

In mammals, conventional B cells, also designated as follicular B cells or B2 cells, produce high affinity antibodies in response to specific epitopes through a somatically recombined B cell receptor (BCR) and, to fulfill this, they require the cooperation of T helper cells in germinal centers (GCs) within the lymphoid follicles. These T-dependent responses are quite effective, but temporally delayed, so T-independent (TI) B cell responses are also mounted immediately after pathogen recognition, to produce low-affinity poly specific antibodies, which offer fast protection, especially at the mucosal interfaces. Innate B cells such as B1 cells or marginal zone (MZ) B cells are mostly responsible for these TI responses (1, 2). Exemplifying further the crosstalk that exists between the adaptive and the innate immune system, B cells (both B2 and innate B cells) receive additional stimulatory signals from other cells of the innate immune system such as dendritic cells (DCs), macrophages, or granulocytes. These innate cells, upon sensing of conserved pathogen features through invariant pattern recognition receptors (PRRs), release B cell-stimulating factors (3, 4) that elicit extrafollicular TI responses (5) or complement the activity of T helper cells in GCs (6).

B cell-activating factor (BAFF) of the tumor necrosis factor (TNF) family (also known as BlyS, TALL-1, THANK, zTNF4, and TNFSF13B) and a proliferation-inducing ligand (APRIL), members of the TNF family, are two of the most important factors among these innate B cell-stimulating cytokines (7). Both of them are type II transmembrane proteins, which become soluble ligands after cleavage at the cell surface by a furin-like protease, with both the membrane-bound and soluble forms being biologically active (8). Concerning the receptors through which they signal, both BAFF and APRIL bind to B cell maturation antigen [(BCMA), also known as TNFRSF17] and transmembrane activator and calcium modulator and cyclophilin ligand interactor (TACI, also known as TNFRSF13B), whereas BAFF also binds to BAFF receptor (BAFF-R, also known as BR3 or TNFRSF13C) (7). All three receptors are preferentially expressed in B cells (9, 10).

In mammals, BAFF is not a stimulator of B cell proliferation on its own, but acts as a potent co-stimulator when combined with BCR engagement (11). On the other hand, BAFF is a strong promoter of B cell survival (12), mainly sustained through BAFF-R signaling (13). For this, BAFF-mediated NF-κB activation has been linked to increased expression of antiapoptotic proteins [reviewed in Ref. (14)]. On the other hand, TACI expression is highly inducible and has been shown to be particularly highly expressed in innate B1 cells (13) and consequently TACI^−/−^ animals are unable to mount normal TI immune responses (15), as the activation and survival of plasmablasts derived from innate B cells is compromised (16). Regarding BCMA, BAFF signaling through this receptor has been proven to be essential for the survival of long-lived plasma cells (17). BCMA can also promote the antigen-presenting function of B cells through upregulation of MHC II and co-stimulatory molecules (18), although BAFF-mediated upregulation of MHC class II expression is also achieved by signaling through TACI and BAFF-R (19).

The sequences of BAFF and APRIL have been previously identified in rainbow trout (*Oncorhynchus mykiss*) (20), along with that of a further fish-specific molecule with close homology to both BAFF and APRIL designated as BALM (BAFF- and APRIL-like molecule). In addition, BAFF sequences have been reported in different teleost fish species including zebrafish (*Danio rerio*) (21), mefugu (*Takifugu obscurus*) (22), Japanese sea perch (*Lateolabrax japonicus*) (23), grass carp (*Ctenopharyngodon idella*) (24), yellow grouper (*Epinephelus awoara*) (25), miiuy croaker (*Miichthys miiuy*) (26), tongue sole (*Cynoglossus semilaevis*) (27), Nile tilapia (*Oreochromis niloticus*) (28), rock bream (*Oplegnathus fasciatus*) (29), and cartilaginous fish such as white-spotted catshark (*Chiloscyllium plagiosum*) (30), spiny dogfish (*Squalus acanthias*) (31), and small-spotted catshark (*Scyliorhinus canicula*) (32). Although recombinant BAFF proteins have been produced for some of these species (21–23, 25, 27–30), many of these studies have exclusively tested the effect of recombinant fish BAFF on B cell survival using mammalian B cells (21, 22, 25, 28, 30). Furthermore, those studies, which have concluded a capacity of BAFF to induce proliferation using teleost leukocytes, have not made a distinction between proliferation and survival as they have only determined increased numbers of leukocytes after BAFF treatment and have not clearly established that the cells showing increased survival or proliferating were in fact B cells (21–23, 27, 29). Consequently, the role that BAFF plays on B cell function in teleost remains largely unknown.

Although, evolutionarily, fish constitute the first group of animals in which all the key components of adaptive immunity are present, some unique structural features of the immune system predict important functional differences between fish and mammalian B cells. In teleost, the spleen constitutes the main secondary immune organ in the absence of lymph nodes. However, the splenic white pulp is poorly developed in comparison to mammals and no GCs are apparent (33). Additionally, fish contain only three immunoglobulin classes, namely IgM, IgD, and IgT (designated as IgZ in some species) (34). Since IgT is a teleost fish-specific Ig that seems to be specialized in mucosal immunity (35, 36) and IgT^+^ B cells constitute a distinct linage (35), no class switch recombination has ever been reported in fish. Furthermore, teleost B cells share many features of mammalian B1 cells, as for example, long-term survival in cell culture, a high phagocytic capacity (37, 38), constitutive expression of many PRRs (39, 40), and expression of homolog molecules to B1-specific cell markers (41). As a result, the lack of teleost follicular structures and the conservation of many innate features of fish B cells strongly suggest that fish B cell responses best resemble mammalian TI responses.

In this context, it is of great interest to establish how innate B cell stimulating cytokines, such as BAFF, control B cells before the appearance of follicular structures and this is what we have addressed in the current study using rainbow trout as a model. Our findings show that, in teleost, as in mammals, BAFF has no effect on B cell proliferation but is exclusively a survival factor for splenic B cells. Interestingly, both splenic IgD^+^IgM^+^ cells (exhibiting a naïve B cell phenotype) and IgD^−^IgM^+^ cells (exhibiting a plasmablast phenotype) are expanded in the presence of BAFF and, consequently, IgM secretion is also augmented in these cultures. BAFF also increased the surface expression of MHC II in splenic B cells, but had no effect on their phagocytic capacity. Finally, we have established that, unlike the situation in mammals where BAFF is produced by cells of the innate immune system, a subpopulation of fish splenic B cells produce BAFF in physiological conditions, revealing an ancient mechanism to regulate B cell functionality in teleost. Throughout evolution, this capacity of B cells to produce BAFF has been lost, possibly upon the appearance of follicular structures that sustain B2 cell survival in physiological conditions, and only reappears in mammals during B cell proliferating disorders such as B cell lymphomas or autoimmune diseases.

## Materials and Methods

### Production of Recombinant Rainbow Trout BAFF

The nucleotide sequence corresponding to the extracellular domain of the rainbow trout BAFF sequence (GenBank Accession number DQ218467.1) together with an N-terminal 6x histidine tag was synthetized and subcloned into the E3 expression vector (Abyntek). The recombinant plasmid was transformed into BL21 cells and a kanamycin-resistant single positive colony was then incubated at 37°C in Luria-Bertani media. When the OD_600_ reached 0.6, 0.1 mM of isopropyl β-d-thiogalactoside (IPTG, Sigma-Aldrich) was added to induce protein production. After 16 h, cells were harvested, lysed by sonication, and dissolved using urea. Thereafter, BAFF was obtained through the use of Nickel columns (Sigma-Aldrich). The BAFF-containing fractions were pooled, refolded, filtered through 0.22 μm, and resuspended in storage buffer (50 mM Tris–HCl, 150 mM NaCl, 10% glycerol, 0.5 M l-arginine, and 2 mM DTT, pH 8.5). Protein concentration was determined in a BCA protein assay (Thermo Fisher Scientific) and the recombinant rainbow trout BAFF (0.3 mg/ml) was aliquoted and stored at −80°C until used. An irrelevant protein with a similar molecular weight to that of recombinant BAFF (20.7 kDa), also bearing an N-terminal His tag was produced in the same conditions (C-His) and was used as a functional control.

### Generation of a Mouse pAb against Rainbow Trout BAFF

Six-week-old BALBc mice were immunized with 60 μg of recombinant BAFF protein emulsified in Montanide ISA 763 A VG adjuvant (Seppic) at a 1:1 ratio (volume). A 60 μg booster shot emulsified in Montanide ISA 763 A VG was given at days 15 and 30 postimmunization. Mice were sacrificed 10 days after the last boost and peripheral blood was collected and serum obtained. The antibody was purified from the serum by immunoglobulin affinity using protein G-sepharose columns (Thermo Fisher Scientific), following manufacturer’s instructions. Recombinant rainbow trout BAFF protein was used to test the specificity of the anti-BAFF polyclonal antibody against the pre-immune serum from the same animal, by Western blot. SDS-PAGE and Western blots were performed as previously described (42). For some applications, purified anti-BAFF antibody was biotinylated using EZ-link NHS Biotin (Thermo Fisher Scientific), following manufacturer’s instructions. The protocol described for the generation of the polyclonal antibody described comply with the Guidelines of the European Union Council (2010/63/EU) for the use of laboratory animals and were previously approved by the Ethics committee from the Instituto Nacional de Investigación y Tecnología Agraria y Alimentaria (INIA; Code CEEA 2012/008).

### Experimental Fish

Healthy specimens of female rainbow trout (*O. mykiss*) of approximately 50–70 g were obtained from Centro de Acuicultura El Molino (Madrid, Spain). Fish were maintained at the Animal Health Research Center (CISA-INIA) laboratory at 16°C with a re-circulating water system and 12:12-h light:dark photoperiod. Fish were fed twice a day with a commercial diet (Skretting, Spain). Prior to any experimental procedure, fish were acclimatized to laboratory conditions for 2 weeks and, during this period, no clinical signs were ever observed. The experiments described comply with the Guidelines of the European Union Council (2010/63/EU) for the use of laboratory animals and were previously approved by the Ethics committee from the Instituto Nacional de Investigación y Tecnología Agraria y Alimentaria (INIA; Code CEEA 2011/044).

### Leukocyte Isolation

Rainbow trout were killed by benzocaine (Sigma-Aldrich) overdose and blood was extracted with a heparinized needle from the caudal vein and diluted 10 times with Leibovitz medium (L-15, Invitrogen) supplemented with 100 IU/ml penicillin together with 100 μg/ml streptomycin (P/S, Life Technologies), 10 U/ml heparin (Sigma-Aldrich), and 5% fetal calf serum (FCS, Life Technologies). Spleen, head kidney, hindgut, skin, and peritoneal exudates were then collected. Single cell suspensions from spleen, head kidney, and peritoneum were obtained using 100 μm nylon cell strainers (BD Biosciences). The hindgut samples were opened lengthwise, washed in phosphate-buffered saline (PBS), and cut into small pieces. The skin was cut off the fish with a scalpel, then, the muscle tissue was removed and the skin was further cleaned with ice-cold PBS and cut into small pieces. For hindgut and skin, the cell extraction procedure started with one round of 30 min agitation at 4°C in L-15 medium with P/S and 5% FCS, followed by an agitation in PBS with 1 mM EDTA and 1 mM DTT for 30 min. Finally, the tissues were digested with 0.15 mg/ml of collagenase (Sigma) in L-15 for 1.5 h at 20°C. All cell suspensions were placed onto 30/51% discontinuous Percoll (GE Healthcare) density gradients and centrifuged at 500 × *g* for 30 min at 4°C. The interface cells were collected and washed twice in L-15 containing 5% FCS. When required, leukocytes were incubated in the presence of TNP-LPS (Biotools) at a final concentration of 5 μg/ml and/or recombinant rainbow trout BAFF at a final concentration of 3 μg/ml. A wide range of doses of these stimuli were tested and the optimal doses were selected based on their effect on B cell survival (data not shown).

### Flow Cytometry

The anti-trout IgD [mAb mouse IgG_1_ coupled to R-phycoerythrin (R-PE), 5 μg/ml], the anti-trout IgM [1.14 mAb mouse IgG_1_ coupled to fluorescein (FITC) or to allophycocyanin (APC), 1 μg/ml], and the anti-trout MHC II β-chain (mAb mouse IgG_1_ coupled to APC, 2 μg/ml) used in this study have been previously characterized (43, 44). All the mAbs were fluorescently labeled using fluorescein, R-PE, or APC Lightning-Link labeling kits (InnovaBiosciences), following manufacturer’s instructions. Spleen leukocytes were incubated with specific antibodies for 30 min in the case of anti-IgM or anti-MHC, or 45 min in the case of anti-IgD, washed three times with staining buffer (PBS containing 1% FCS and 0.5% sodium azide), and analyzed. A biotinylated version of anti-BAFF (pAb mouse IgG, 1 μg/ml) was used to determine endogenous BAFF expression by leukocytes. To carry this out, cells were incubated for 30 min with biotinylated anti-BAFF pAb, then washed three times with staining buffer, and incubated for another 30 min with streptavidin-FITC (Thermo Fisher Scientific). In all cases, isotype controls for mouse mAbs and anti-BAFF pAb (BD Biosciences) were tested in parallel to discard unspecific binding of the Abs. All the incubations were performed at 4°C. After incubation with the corresponding stimuli, samples were incubated with 10 μg/ml propidium iodide (Thermo Fisher) for 5 min in the dark, and cell viability was analyzed in our experimental conditions (*n* = 3). All samples were analyzed on a FACSCalibur flow cytometer (BD Biosciences) equipped with CellQuest Pro software. Flow cytometry analysis was performed with FlowJo 10 (TreeStar).

### BAFF-Binding Assay

For the analysis of BAFF binding to trout B cells, splenocytes were incubated for 1 h at 4°C with recombinant BAFF or C-His (3 μg/ml each) or with a stock of BAFF, which had been previously incubated with anti-BAFF pAb (protein:Ab molar ratio 1:10). After that, cells were washed with staining buffer and labeled with an FITC-anti-His mAb (Thermo Fisher Scientific) together with APC-anti-IgM mAb (1 μg/ml) for 30 min at 4°C, then washed again, and analyzed by flow cytometry.

### Tissue Sampling for the Analysis of BAFF Transcription

Rainbow trouts were killed by benzocaine overdose. Blood was extracted with a heparinized needle from the caudal vein. Spleen, head kidney, skin, heart, gills, thymus, hindgut, and liver were then collected and placed in Trizol (Thermo Fisher Scientific) after transcardial perfusion using teleost Ringer solution pH 7.4 with 0.1% procaine in order to remove all the circulating blood from the tissues (39, 45). Total RNA was extracted using a combination of Trizol and RNAeasy Mini kit (Qiagen) as previously described (43). RNA was also extracted from the RTS11 rainbow trout monocyte–macrophage cell line (46) and the RTG2 fibroblast cell line (ATCC CCL-55) following the same procedure. The expression of individual genes was normalized to relative expression of trout EF-1α and the expression levels were calculated using the 2^−ΔCt^ method, where ΔCt is determined by subtracting the EF-1α value from Ct of the targeted gene as previously described (43). RNA pellets were eluted from the columns in RNase-free water, quantified in a Nanodrop 1000 spectrophotometer (Thermo Scientific), and stored at −80°C until used. RNAs were treated with DNase during the purification process to remove genomic DNA that might interfere with the PCR reactions and then used to obtain cDNA in each sample using the Bioscript reverse transcriptase (Bioline Reagents Ltd.) and oligo (dT)_12–18_ (0.5 μg/ml) following manufacturer’s instructions. Minus reverse transcriptase controls were included in all the assays to rule out amplification of genomic DNA. The resulting cDNA was diluted 1:5 in DNAse-free water and stored at −20°C. Transcription levels were analyzed by real-time PCR with a LightCycler^®^ 96 System (Roche) using FastStart Essential DNA Green Master reagents (Roche) and specific primers (Table S1 in Supplementary Material). Each sample was measured under the following conditions: 10 min at 95°C, followed by 40 amplification cycles (10 s at 95°C, 10 s at 60°C, and 10 s at 72°C). The expression of individual genes was normalized to relative expression of trout EF-1α and the expression levels were calculated using the 2^−ΔCt^ method, where ΔCt is determined by subtracting the EF-1α value from Ct of the targeted gene as previously described (43). Negative controls with no template were included in all the experiments. A melting curve for each PCR was determined by reading fluorescence every degree between 60 and 95°C to ensure that only a single product had been amplified.

### Gene Expression Analysis in FACS-Isolated Populations

To analyze BAFF transcription levels, IgM^+^ B cells were isolated from spleen, head kidney, blood, and peritoneum. To carry this out, cells were FACS isolated with specific mAbs against IgM, as previously described (39). CD8^+^ T cells (CD8^+^MHC II^−^ cells) were isolated from spleen, and CD8^+^ DCs (myeloid CD8^+^MHC II^+^) were obtained from skin using specific mAbs against CD8 and MHC II, as previously described (44). To analyze the gene expression pattern of B cell subsets from the spleen, splenocytes were stained using mAbs against IgD and IgM, as described above, and IgD^+^IgM^+^ and IgD^−^IgM^+^ B cells were FACS isolated. To analyze the transcriptional levels of MHC II and Blimp-1 on BAFF-treated B cells, splenocytes were stained using a mAb against IgM, as described above, and IgM^+^ B cells were FACS isolated. In all cases, FACS isolation was performed using a BD FACSAria III (BD Biosciences). We analyzed the purity of the isolated populations by flow cytometry after sorting and only those samples showing a purity level higher than 95% were used for qPCR analysis. RNA isolation and expression of individual genes was performed as previously described (44). Briefly, total RNA from FACS-isolated cells was isolated using the Power SYBR Green Cells-to-Ct Kit (Thermo Fisher Scientific) following manufacturer’s instructions. RNAs were treated with DNase during the process to remove genomic DNA that might interfere with the PCR reactions. Reverse transcription was also performed using the Power SYBR Green Cells-to-Ct Kit according to manufacturer’s instructions. Minus reverse transcriptase controls were included in all the assays to rule out amplification of genomic DNA. To evaluate the levels of transcription of BAFF in these samples, real-time PCR was performed with a LightCycler^®^ 96 System instrument using SYBR Green PCR core Reagents (Applied Biosystems) and specific primers (Table S1 in Supplementary Material). Each sample was measured in duplicate under the following conditions: 10 min at 95°C, followed by 45 amplification cycles (15 s at 95°C and 1 min at 60°C). A melting curve for each PCR was also included to ensure only a single product had been amplified. The expression of individual genes was normalized to the relative expression of trout housekeeping gene EF-1α elongation factor as described above.

### Phagocytic Activity

For the analysis of phagocytosis, spleen leukocytes were seeded in 24-well plates (Nunc) at a cell density of 1 × 10^6^ cells per well and incubated for 24 h at 20°C with BAFF (3 μg/ml), TNP-LPS (5 μg/ml), a combination of both, or left unstimulated (control), and then incubated for another 16 h at 20°C with fluorescent polystyrene-based particles (FluoSpheres^®^ Microspheres, 1.0 μm, Crimson Red Fluorescent 625/645, 2% solids; Life Technologies) at a cell:bead ratio of 1:10 or without beads as negative controls. Cells were harvested using a standard cell scraper (Corning). Non-ingested beads were removed by centrifugation (100 × *g* for 10 min at 4°C) over a cushion of 3% (weight/volume) bovine serum albumin [(BSA), Fisher Scientific] in PBS supplemented with 4.5% (weight/volume) d-glucose (Sigma). Cells were resuspended in staining buffer, labeled with an anti-IgM mAb fluorescently labeled with FITC, and analyzed on a FACSCalibur flow cytometer equipped with CellQuest sofware (BD Biosciences). The analysis was also performed with FlowJo 10.

### B Cell Proliferation

The BrdU Flow Kit (Becton Dickinson) was used to measure the proliferation of IgM^+^ B cells in response to BAFF and/or TNP-LPS following manufacturer’s instructions. Splenocytes at a concentration of 2 × 10^6^ cells/ml were incubated for 3 days at 20°C with the stimuli as described above. Bromodeoxyuridine (BrdU, 10 μM) was then added to the cultures and the cells were incubated for an additional 24 h. The cells were collected and stained with APC-anti-IgM mAb (1 μg/ml). Briefly, to analyze incorporation of BrdU, cells were then fixed and permeabilized with Cytofix/Cytoperm Buffer for 15 min on ice, then incubated with Cytoperm Permeabilization Buffer Plus for 10 min on ice, and re-fixed with Cytofix/Cytoperm Buffer for 5 min at RT. Cells were then incubated with DNase (30 μg/10^6^ cells) for 1 h at 37°C to expose the incorporated BrdU. Finally, cells were stained with FITC anti-BrdU antibody for 20 min at RT and analyzed by flow cytometry.

### Confocal Microscopy

Splenocytes were obtained as described above. To establish BAFF binding to trout IgM^+^ B cells, leukocytes were incubated with 3 μg/ml of recombinant BAFF in L-15 media supplemented with 5% FCS. After 1 h at 20°C, the cells were washed with serum-free L-15 medium, seeded on poly l-lysine coated slides, and incubated at 20°C for 30 min. After gently washing with PBS, the slides were fixed in 4% PFA for 15 min at RT. The samples were permeabilized for 1 h at RT with permeabilizing solution (TBS buffer, pH = 7.5, containing 0.01% BSA, 0.02% Tween-20, and 0.5% saponin) and then incubated for 1 h at RT with blocking solution (TBS buffer, pH = 7.5, containing 0.01% BSA, 0.02% Tween-20, 0.5% saponin, and 10% rabbit and goat serum). Fixed cell slides were then incubated with APC-anti-IgM mAb (1 μg/ml) and an FITC-anti-His mAb for 30 min. Samples were counterstained with 1 μg/ml DAPI (Sigma).

To analyze BAFF production by IgM^+^ B cells, leukocytes were suspended in serum-free L-15 medium, seeded on poly l-lysine coated slides, and incubated at 20°C for 30 min. After gently washing with PBS, the slides were fixed, permeabilized, and washed as described above. Fixed cell slides were then incubated with anti-BAFF pAb coupled to biotin for 30 min, then gently washed, and incubated with APC-anti-IgM (1 μg/ml) and streptavidin-FITC (Thermo Fisher Scientific) for 30 min. Samples were counterstained with 1 μg/ml DAPI (Sigma). In all cases, laser scanning confocal microscopy images (0.3 μm thickness) were acquired with an inverted Zeiss Axiovert LSM 880 microscope. Images were analyzed with Zen 2.0 (Carl Zeiss) and Fiji (NIH) software packages.

### ELISPOT Analysis

ELISPOT was used to quantify the number of IgM-secreting B cells. Splenocytes were incubated with BAFF (3 μg/ml), and/or TNP-LPS (5 μg/ml), or left unstimulated (control) at 20°C for 48 h. ELISPOT plates containing Inmobilon-P membranes (Millipore) were activated with 70% ethanol for 30 s, coated with anti-trout IgM mAb (clone 4C10) at 2 μg/ml in PBS, and incubated overnight at 4°C. To block non-specific binding to the membrane, plates were then incubated with 2% BSA in PBS for 2 h at RT. Thereafter, leukocytes from individual fish were added to the wells in triplicate at a concentration of 1 × 10^5^ cells per well. After 24 h of incubation at 20°C, cells were washed away five times with PBS and plates were blocked again with 2% BSA in PBS for 1 h at RT. After blocking, biotinylated anti-trout IgM mAb (clone 4C10) was added to the plates and incubated at 1 μg/ml for 1 h at RT. Following additional washing steps (five times in PBS), the plates were developed using streptavidin-HRP (Thermo Scientific) at RT for 1 h, washed again with PBS, and incubated with 3-amino 9-ethylcarbazole (Sigma-Aldrich) for 30 min at RT in the dark. Substrate reaction was stopped by washing the plates with tap water. Once the membranes had dried, they were digitally scanned and spot counts determined by the ImmunoSpot Series 45 Micro ELISPOT Analyzer.

### Statistical Analysis

Statistical analyses were performed conducting one-way ANOVA to analyze whether significant differences exist between the means of all the experimental groups included in each assay. When significant differences were found, we then performed a *post hoc* multiple comparison Tukey’s test. The differences between the mean values were considered significant on different degrees, where *p ≤ 0.05, **p ≤ 0.01, and ***p ≤ 0.005. The analysis was performed with XLSTAT statistical software for Excel (Microsoft) and GraphPad Prism 6 software (GraphPad).

## Results

### Recombinant BAFF Binds Teleost B Cells

The soluble domain of rainbow trout BAFF was produced in *E. coli*, purified under denaturing conditions, and refolded *in vitro* (Figure S1A in Supplementary Material). To confirm that LPS contamination in the recombinant protein was negligible, we verified that recombinant BAFF had no effects on the expression of IL-1β on splenocytes, since this gene has been shown to be highly upregulated by bacterial LPS in this system (47, 48) (Figure S1B in Supplementary Material).

**Figure 1:**
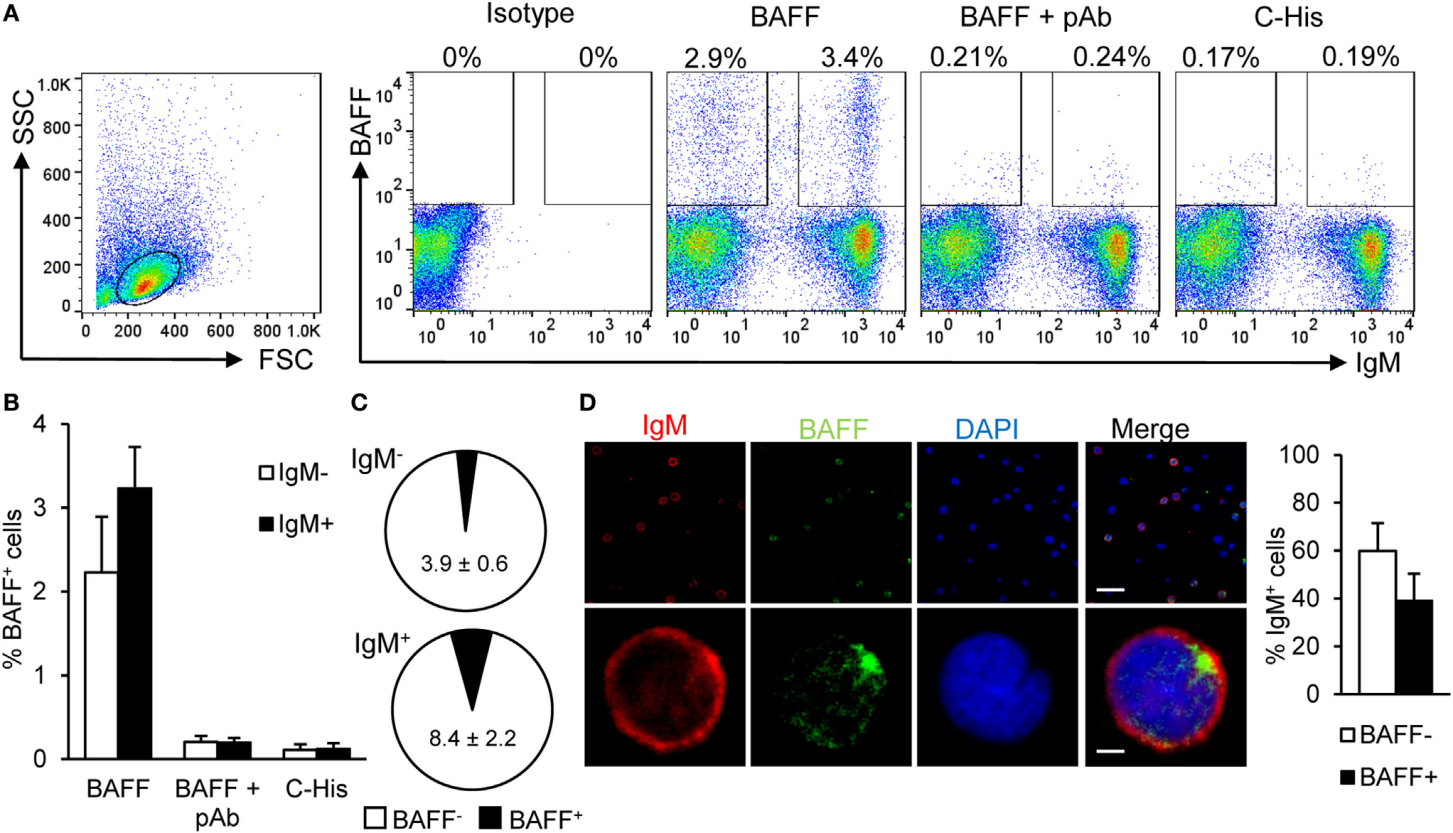
**Rainbow trout B cell-activating factor (BAFF) binds to IgM^+^ B cells**. To analyze the specific binding of BAFF to IgM^+^ B cells, freshly isolated splenic leukocytes were incubated with recombinant histidine-tagged BAFF protein (3 μg/ml) or an irrelevant 20 kDA histidine-tagged protein (C-His, 3 μg/ml) for 1 h. In parallel, cells were also cultured for 1 h with recombinant BAFF protein, which had been previously incubated for 1 h with an anti-BAFF pAb (molar ratio 1:10). Thereafter, cells were stained with an anti-IgM mAb together with an anti-His mAb, or with isotype control mAbs, and analyzed by flow cytometry. Dot plots from one representative experiment are shown **(A)** and percentages of BAFF-binding IgM^−^ and IgM^+^ cells are indicated in the plots. The quantification of BAFF binding on IgM^−^ and IgM^+^ B cells is also shown **(B)** as mean + SD (*n* = 9, three experiments containing three animals each). **(C)** The percentages of cells binding BAFF within the IgM^−^ and IgM^+^ compartments were also calculated and are plotted as pie charts (mean ± SD, *n* = 9, three independent experiments containing three animals each). Dead cells were not excluded during the flow cytometry analysis, but cell viability was higher than 99% for IgM^+^ B cells and higher than 95% for other lymphocytes on these experimental settings (Figure S4A in Supplementary Material). **(D)** Total leukocytes from spleen were incubated with recombinant BAFF (3 μg/ml) for 1 h, then plated onto poly-l-lysine-coated glass slides, fixed and labeled with anti-IgM (shown as red) and anti-His (BAFF, shown as green) antibodies, then counterstained with DAPI (blue), and analyzed by fluorescence microscopy. A representative general overview is shown (upper row) (scale bar, 20 μm) and the amplification detail of a single cell (lower row) (scale bar, 1 μm). IgM^+^BAFF^−^ and IgM^+^BAFF^+^ cells on preparations of spleen leukocytes from eight fish were quantified and plotted (right bar plot) and shown as mean + SD (*n* = 150 cells).

Next, we verified that splenic IgM^+^ B cells were able to bind recombinant BAFF. For this, we incubated total leukocytes from the spleen of unstimulated fish with recombinant histidine-tagged BAFF for 1 h, then labeled the cells with an anti-IgM mAb and an anti-His mAb, and performed flow cytometry analysis. We verified that an average 6.5% of the splenic lymphocytes-bound recombinant BAFF and more than half of these cells were IgM^+^ B cells (Figures 1A,B). These BAFF-binding IgM^+^ B cells correspond to an average 8.4% of the IgM^+^ B cell population, whereas only 3.9% of the IgM^−^ B cells were binding the recombinant cytokine (Figure 1C). To confirm that BAFF binding to B cells was specific, we blocked the union with a polyclonal antibody raised against recombinant trout BAFF in mice. This polyclonal antibody was highly specific and recognized recombinant BAFF protein in Western blot (Figure S2A in Supplementary Material) and also membrane BAFF present on spleen lymphocytes by flow cytometry (Figure S2B in Supplementary Material). In the splenocyte BAFF-binding assays, when recombinant BAFF was incubated with the polyclonal anti-BAFF before its addition to splenocytes, its binding decreased to nearly undetectable levels, both in IgM^+^ and IgM^−^ populations (Figures 1A,B), demonstrating the specificity of the binding and that of the polyclonal antibody. To rule out a possible binding of BAFF through the histidine tag in an unspecific fashion, we incubated splenocytes with an irrelevant protein of the same molecular weight carrying a histidine tag, and as shown in Figures 1A,B, only a negligible binding was observed. An additional control was included to exclude the possibility of the anti-BAFF pAb blocking the access of the anti-His mAb to the His tag instead of BAFF binding to the cells, we incubated spleen leukocytes with BAFF for 1 h, and then incubated these cells with anti-BAFF pAb for 1 h. In this case, we did not see any difference on BAFF binding, thus demonstrating that the pAb anti-BAFF blocks the binding of BAFF to the cells but does not interfere with the access of the anti-His to the His tag once BAFF is bound to the cells (Figure S3 in Supplementary Material). To visualize BAFF binding to IgM^+^ B cells, we incubated total leukocytes from the spleen of unstimulated fish with recombinant histidine-tagged BAFF for 1 h, and then fixed the cells to analyze them through confocal microscopy. We further verified that spleen IgM^+^ B cells co-stained with recombinant BAFF (Figure 1D). In this case, the average number of BAFF^+^ cells observed within the IgM^+^ compartment was 39.2%, which was higher than that seen for extracellular binding analyzed by flow cytometry. The reason for this difference is that, through confocal microscopy, we are able to observe both extracellular (binding) and intracellular (internalization) staining, however, we cannot exclude that part of the internalization of BAFF is due to receptor-independent mechanisms such as pinocytosis. All together, these results unequivocally demonstrate that a subpopulation of trout splenic B cells is able to bind to soluble BAFF.

### Spleen B Cells Express BAFF Constitutively in Teleost

**Figure 2:**
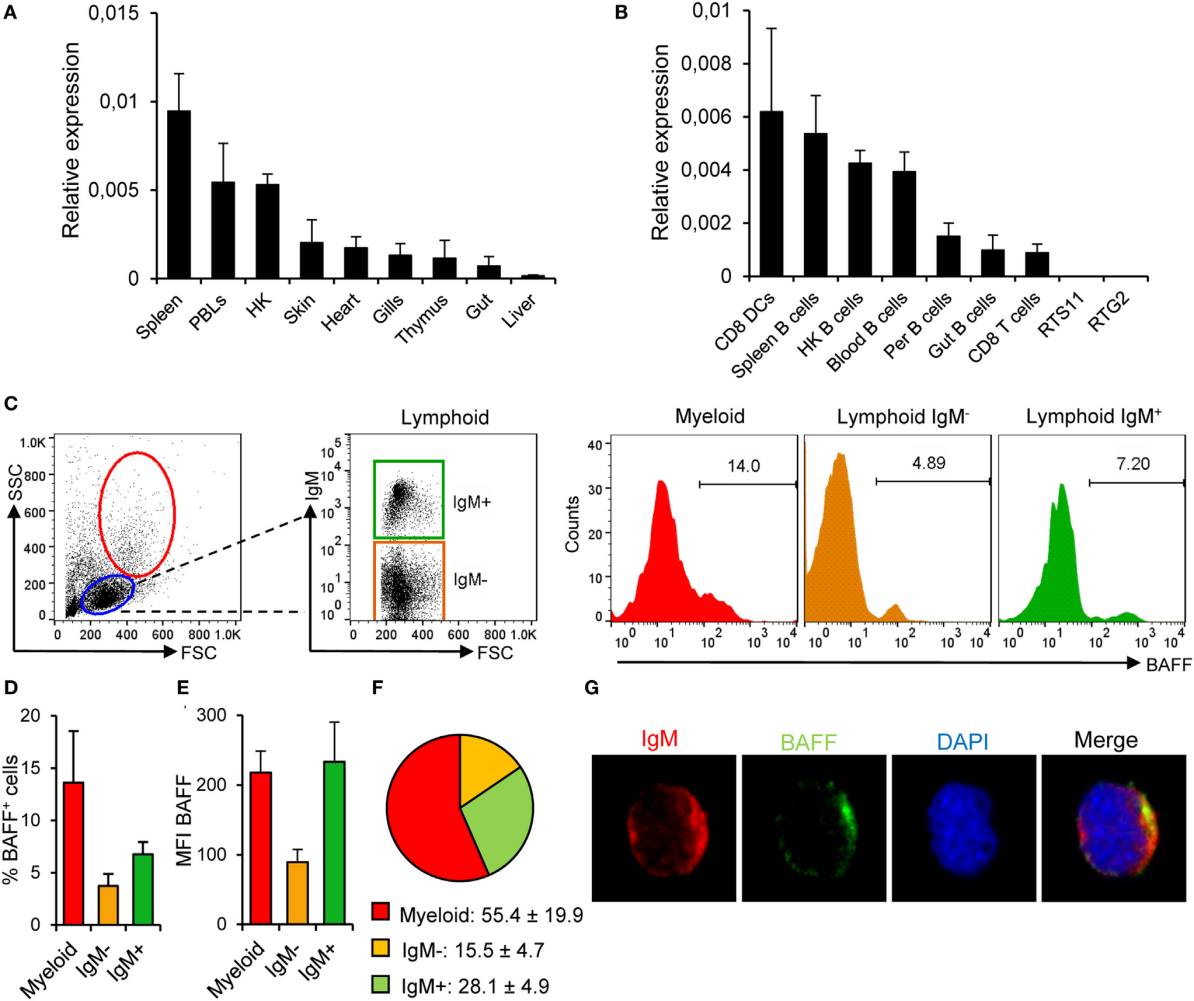
**B cell-activating factor (BAFF) expression in rainbow trout in physiological conditions**. BAFF transcription levels were analyzed through real-time PCR in rainbow trout tissues **(A)** and FACS isolated cell subsets and in the RTS11 (macrophages) and RTG2 (fibroblasts) cell lines **(B)** the relative expression to the endogenous control EF-1α was calculated for each sample (shown as mean + SD, *n* = 6) (HK, head kidney; DCs, dendritic cells; Per, peritoneum). **(C)** Rainbow trout leukocytes isolated from spleen were stained with an anti-IgM mAb together with an anti-BAFF pAb and analyzed by flow cytometry. Lymphoid (blue gate) and myeloid (red gate) populations were gated and, then, IgM^−^ (orange gate) and IgM^+^ (green gate) cells were further selected from the lymphoid population. A representative histogram showing the level of BAFF expression in each population is shown. The average percentage of BAFF^+^ cells within each compartment was also calculated **(D)** as well as the mean fluorescence intensity of BAFF on those populations **(E)** (shown as mean + SD, *n* = 9, three independent experiments containing three animals each). **(F)** The percentages of myeloid, lymphoid IgM^−^, and lymphoid IgM^+^ cells within the BAFF^+^ compartment were also calculated and are plotted as pie charts (mean ± SD, *n* = 9, three independent experiments containing three animals each). Dead cells were not excluded during the flow cytometry analysis, but cell viability was higher than 99% for IgM^+^ B cells and higher than 95% for other lymphocytes on these experimental settings (Figure S4B in Supplementary Material). **(G)** Total leukocytes from spleen were plated onto poly-l-lysine-coated glass slides, fixed and labeled with anti-IgM (red) and anti-BAFF (green) antibodies, then counterstained with DAPI (blue), and analyzed by fluorescence microscopy. Images from one representative experiment are shown (scale bar, 1 μm).

Prior to the determination of BAFF functionality in trout B cells, we conducted a series of experiments to study, which cell types were producing BAFF in physiological conditions. First, we analyzed the transcription of BAFF throughout the different tissues in unstimulated rainbow trout. BAFF mRNA levels were higher in the spleen, followed by other immune tissues such as peripheral blood and the head kidney (the main hematopoietic organ in fish) (Figure 2A). Intermediate BAFF transcript levels were observed in skin, heart, gills, thymus, and gut, while very low transcript levels were found in the liver (Figure 2A). BAFF transcription was then examined in different immune cell subsets. As expected, CD8^+^ DCs from skin expressed high levels of BAFF (Figure 2B), but to our surprise, these levels were comparable to those observed in IgM^+^ B cells from spleen, head kidney, and blood (Figure 2B). Intermediate BAFF expression levels were found in IgM^+^ B cells from other peripheral tissues, such as the peritoneal cavity or the hindgut. On the other hand, CD8^+^ T cells from the spleen contained low amounts of BAFF mRNA, while no BAFF transcription was observed in the RTS11 monocyte-macrophage or the RTG2 fibroblast cell lines (Figure 2B).

Because mammalian unstimulated B cells do not express BAFF to confirm the transcriptomic results that suggested that teleost naïve B cells produce this cytokine, we studied the presence of BAFF in spleen IgM^+^ cells through flow cytometry using the anti-BAFF polyclonal antibody developed. Approximately 14% of the cells within the myeloid gate (large size and high complexity) were producing BAFF, which could indicate BAFF production by DCs and macrophages (Figures 2C,D). Within the lymphoid gate, we studied BAFF production in IgM^+^ and IgM^−^ cell populations, verifying that a fraction of IgM^+^ B cells express endogenous BAFF on the cell surface (Figures 2C,D). In average, almost 7% of the splenic IgM^+^ B cells produced BAFF, whereas only around 3.7% of the IgM^−^ lymphocytes produced the cytokine (Figures 2C,D). These results reveal that within the BAFF-producing leukocyte compartment, although the majority of the cells producing BAFF were myeloid cells (55.4 ± 19.9%), 28.1 ± 4.9% of the BAFF-producing cells among splenocytes were IgM^+^ B cells (Figure 2F). Furthermore, the levels of membrane BAFF seen in IgM^+^ B cells, estimated by the mean fluorescence intensity (MFI) values, were equivalent to those found in myeloid cells and were much higher than those seen in IgM^−^ leukocytes (Figure 2E). When the anti-BAFF pAb was premixed with recombinant BAFF, we did not observe any binding of the antibody to either lymphoid or myeloid cells, thus confirming that we were observing a specific staining for membrane BAFF (Figure S2C in Supplementary Material). In order to visualize IgM^+^ B cells-expressing BAFF, splenocytes were fixed and labeled with anti-IgM and anti-BAFF antibodies and analyzed by laser scanning confocal microscopy. We observed that IgM^+^ B cells from non-stimulated animals exhibited a positive staining for BAFF (Figure 2G) in a proportion similar to that seen by flow cytometry. These results demonstrate that teleost spleen B cells differ from mammalian B cells in their capacity to produce BAFF in physiological conditions.

### BAFF Upregulates Membrane MHC-II Expression on B Cells without Altering Their Antigen Acquisition Capacities through Phagocytosis

**Figure 3:**
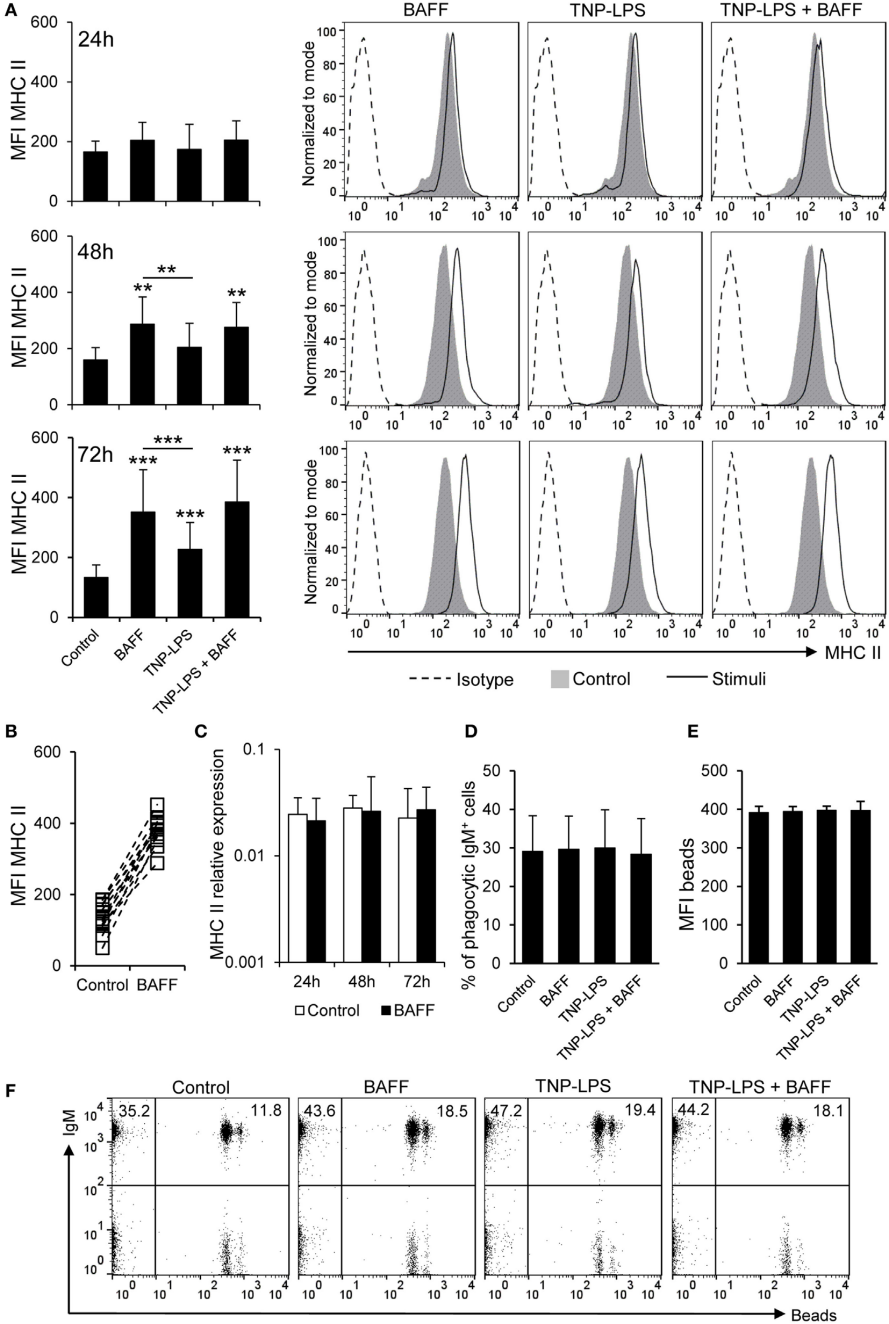
**B cell-activating factor (BAFF) increases the expression of membrane MHC II without altering antigen acquisition by phagocytosis**. **(A)** Spleen leukocytes were incubated with BAFF (3 μg/ml), TNP-LPS (5 μg/ml), a combination of both, or left unstimulated (control) for 24, 48, or 72 h at 20°C. After this time, cells were labeled with anti-IgM and anti-MHC II mAbs and analyzed by flow cytometry. Mean fluorescence intensity (MFI) for membrane MHC II was measured on IgM^+^ B cells and average values were plotted as mean + SD (left) (*n* = 12 from four independent experiments containing three animals each). Histograms from one representative experiment, showing MHC II MFI on IgM^+^ gated B cells are shown (right panels). **(B)** The MFI of MHC II for each individual fish under control or BAFF stimulation conditions for 72 h is also plotted. **(C)** Splenocyte cultures were treated with BAFF (3 μg/ml) or left unstimulated (control) for 24, 48, and 72 h, and then, RNA was extracted from IgM^+^ FACS isolated B cells, as described in Section “Materials and Methods.” The expression of MHC II relative to the endogenous control gene EF-1α was calculated for each sample and shown as mean + SD (*n* = 12, from four independent experiments containing three animals each). Splenocyte cultures were treated with BAFF (3 μg/ml), TNP-LPS (5 μg/ml), or left unstimulated (control) for 48 h, then incubated with Crimson Red fluorescent polystyrene beads (1 μm diameter) in a ratio of 1:10 (cell:beads) for 16 h, and then centrifuged through a 3% bovine serum albumin + 4.5% glucose gradient to remove adhered beads. The percentages of phagocytic cells within the IgM^+^ B cell compartment was determined **(D)** as well as the MFI of the beads internalized by IgM^+^ B cells in each condition **(E)** (*n* = 12, from four independent experiments containing three animals each). Dot plots from one representative experiment for each experimental condition are shown **(F)**. Dead cells were not excluded during the flow cytometry analysis, but cell viability was higher than 99% for IgM^+^ B cells and higher than 95% for other lymphocytes on these experimental settings (Figure S4C in Supplementary Material). Statistical differences were evaluated by a one-way ANOVA followed by a multiple comparison Tukey’s test, where **p ≤ 0.01 and ***p ≤ 0.005.

We studied whether BAFF could have an effect on the levels of surface MHC-II expression using a specific anti-trout MHC II antibody (44). Our results showed that, after incubation with BAFF, membrane MHC II levels increased over time on IgM^+^ B cells reaching a significantly increased peak of expression at 72 h (Figures 3A,B). We compared our results to those elicited by TNP-LPS, given the fact that LPS is a highly effective polyclonal activator of B cells from many species. Although TNP-LPS also upregulated MHC II surface expression, the levels induced by BAFF were significantly higher than those elicited by TNP-LPS and no synergies between TNP-LPS and BAFF were observed (Figure 3A). The increase of membrane MHC II expression was clear in cell cultures from all fish examined individually (Figure 3B) and was not observed when a histidine-tagged irrelevant protein with a similar molecular weight was used (Figure S5A in Supplementary Material).

To assess whether the increase on membrane MHC II level was a consequence of increased gene transcription, we analyzed MHC II transcription levels in sorted IgM^+^ B cells treated or not with recombinant BAFF for 24, 48, and 72 h. We found that MHC II mRNA levels were not upregulated by BAFF in sorted B cells at any time point analyzed (Figure 3C), suggesting that the effect of BAFF could be based on translocation of MHC II molecules to the plasma membrane or an increase of the half-life of membrane MHC II due to a potential activation of B cells (49).

Since rainbow trout B cells have been shown to have potent phagocytic capacities (37), and this peculiar trait strongly conditions its antigen-presenting abilities, we aimed to study whether BAFF could also affect the phagocytic capacity of IgM^+^ B cells. In this occasion, we observed that BAFF did not alter the average percentage of phagocytic IgM^+^ B cells. TNP-LPS or a combination of BAFF with TNP-LPS did not affect the number of phagocytic IgM^+^ B cells either (Figures 3D,F). In parallel, the MFI of beads ingested by the IgM^+^ B cells was not affected by any of the stimuli tested (Figures 3E,F). Thus, our results suggest a role for BAFF in the regulation of B cell antigen presentation, through increased surface MHC II levels but without affecting antigen acquisition by phagocytosis.

### BAFF Has No Proliferative Effects of Trout B Cells

**Figure 4:**
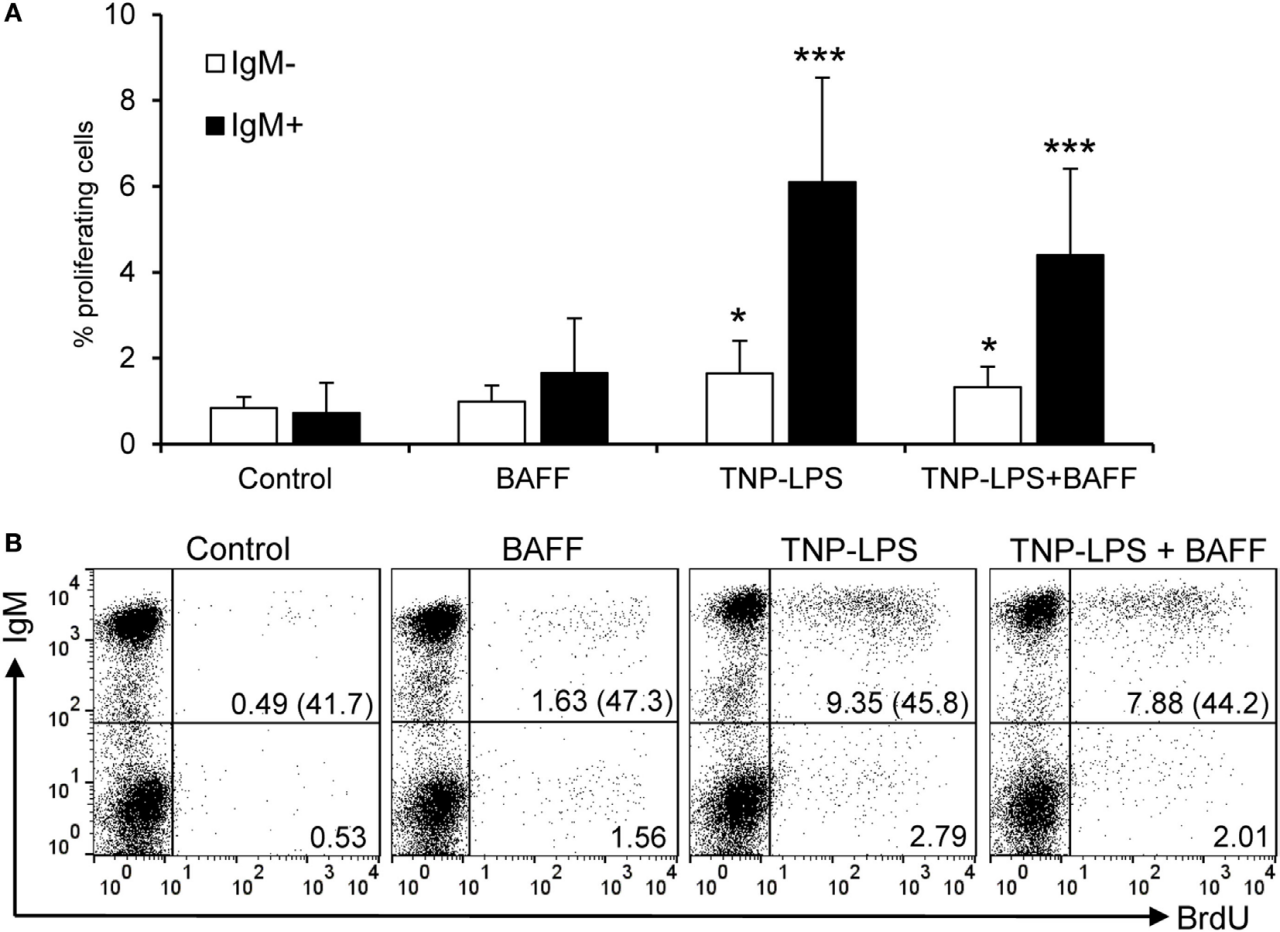
**Rainbow trout B cell-activating factor (BAFF) has no lymphoproliferative effects**. To test the effect of BAFF on IgM^+^ B cell proliferation, spleen leukocytes were incubated with BAFF (3 μg/ml), TNP-LPS (5 μg/ml), a combination of both, or left unstimulated (control) for 3 days at 20°C. After this time, cells were labeled with BrdU and incubated for a further 24 h. The percentage of proliferating (BrdU^+^) IgM^+^ B cells was then determined as described in Section “Materials and Methods.” Quantification of the proliferating IgM^−^ and IgM^+^ populations is shown **(A)** as mean + SD (*n* = 12, from four independent experiments containing three animals each), together with a representative dot plot of the flow cytometry analysis **(B)** number of proliferating IgM^−^ and IgM^+^ cells are also indicated within the dot plots. The numbers between brackets represent the total number of IgM^+^ cells in each case. Dead cells were not excluded during the flow cytometry analysis, but cell viability was higher than 99% for IgM^+^ B cells and higher than 95% for other lymphocytes on these experimental settings (Figure S4C in Supplementary Material). Statistical differences were evaluated by a one-way ANOVA followed by a multiple comparison Tukey’s test, where *p ≤ 0.05 and ***p ≤ 0.005.

A few studies in fish have concluded a capacity of BAFF to induce proliferation of total leukocyte populations using methodological approaches that do not make a distinction between proliferation and survival (22, 23, 27, 29). Thus, in this study, we wanted to clearly address this issue and clarify whether fish BAFF has proliferative effects or, as in mammals, it is exclusively a survival factor. When the proliferative effects of BAFF were tested on IgM^+^ B cells from spleen by means of BrdU incorporation, we verified that BAFF is incapable of inducing proliferation of IgM^+^ B cells, with almost undetectable proliferation rates that were significantly lower than those elicited by TNP-LPS (Figures 4A,B). When TNP-LPS was combined with BAFF, the TNP-LPS-elicited proliferation was not further affected (Figures 4A,B), revealing that BAFF does not even have a synergistic effect on the B cell proliferation induced by TNP-LPS. In a similar way, but a lower extent, TNP-LPS was also able to induce the proliferation of an IgM^−^ lymphocyte population, but this population did not proliferate in response to BAFF either (Figures 4A,B).

### BAFF Is a Survival Factor for Trout IgM^+^ B Cells

**Figure 5:**
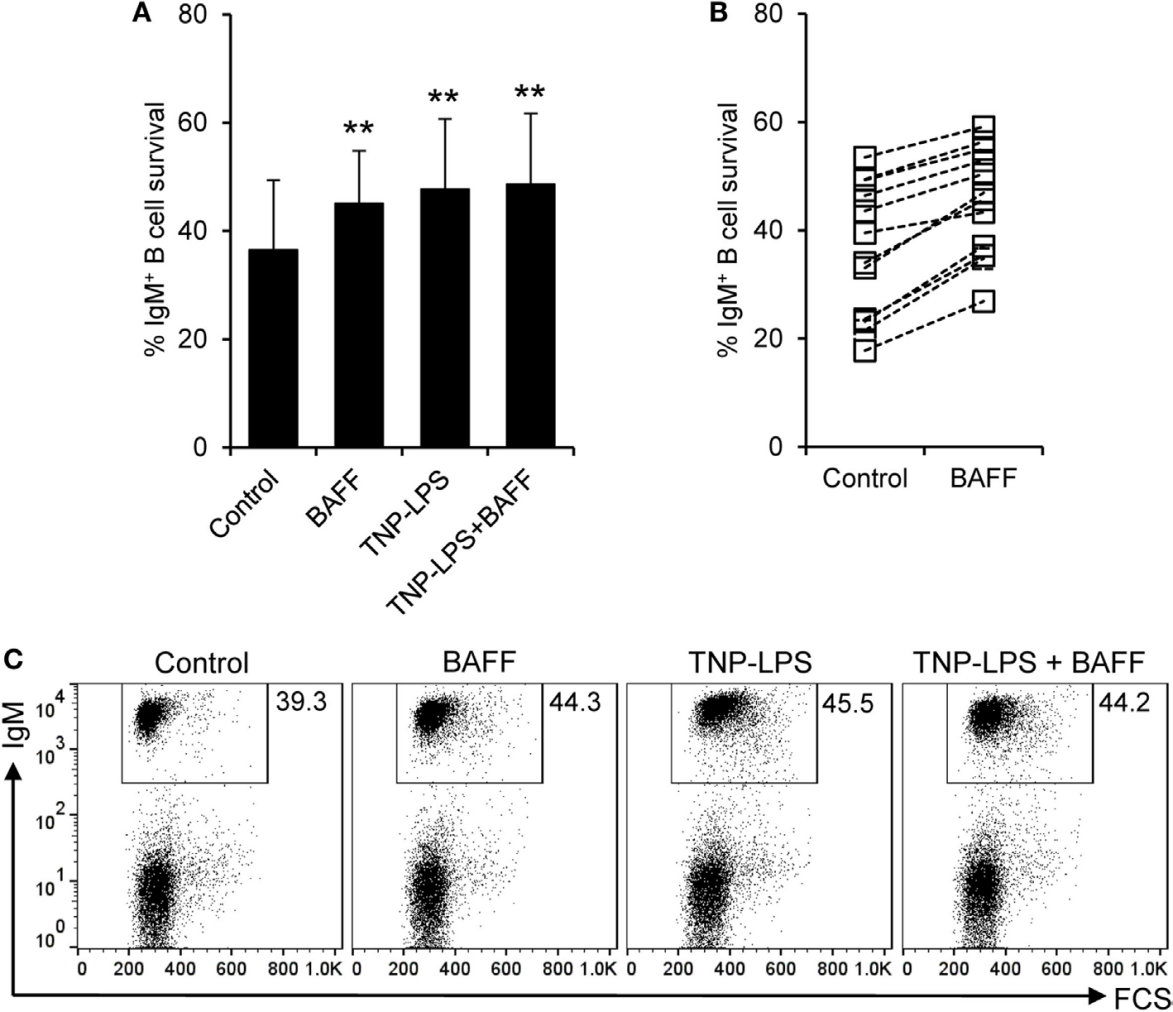
**Rainbow trout B cell-activating factor (BAFF) promotes B cell survival**. Spleen leukocytes were incubated with recombinant BAFF (3 μg/ml), TNP-LPS (5 μg/ml), a combination of both, or left unstimulated (control) for 3 days at 20°C. After this time, cells were labeled with an anti-IgM mAb and analyzed by flow cytometry. The percentage of live IgM^+^ B cells among the lymphocyte gate was then determined. Quantification of average B cell survival is shown as mean + SD **(A)**, as well as the percentage of survival for each individual fish under control or BAFF stimulation conditions **(B)** (*n* = 12, from four independent experiments containing three animals each). A representative dot plot for each experimental condition is also included **(C)**. Dead cells were not excluded during the flow cytometry analysis, but cell viability was higher than 99% for IgM^+^ B cells and higher than 95% for other lymphocytes on these experimental settings (Figure S4C in Supplementary Material). Statistical differences were evaluated by a one-way ANOVA followed by a multiple comparison Tukey’s test, where **p ≤ 0.01.

Despite the negligible proliferative effects, BAFF significantly increased the survival of IgM^+^ B cells compared to control cultures, determined as the percentage of IgM^+^ cells in culture after 3 days, at levels comparable to those induced by TNP-LPS (Figures 5A,C). This increased survival was very consistent and was visualized in cell cultures from all fish examined individually (Figure 5B), but was not observed when an irrelevant protein with a similar weight and a histidine tag was used (Figure S5B in Supplementary Material). Interestingly, no synergistic effects were observed when splenocytes were incubated with BAFF together with TNP-LPS (Figures 5A,C). Although it would be very interesting to study the effect of BAFF on FACS-isolated IgM^+^ B cells, no functional studies can be performed on isolated IgM^+^ B cells, since the only tool available for their isolation is an anti-IgM mAb, which triggers their activation through BCR cross-linking. Thus, to further demonstrate that BAFF had a direct impact on IgM^+^ and to rule out that the increase on the number of IgM^+^ cells mediated by BAFF was due to a negative effect of the cytokine on IgM^−^ cells, we conducted a similar study, this time also analyzing the number of cells at time 0 h. Our experiments revealed that at time 0 h, the cultures contain a lymphocyte population that accounts for an average 81.9 ± 7.8% of the live cells. Although throughout culture, the number of lymphocytes decreased over time, the presence of BAFF in the medium promoted a significant increase in the number of total lymphocytes (Figures S6A,D in Supplementary Material). This increased survival was detected in the IgM^+^ compartment (Figures S6C,E in Supplementary Material) but not in the IgM^−^ (Figures S6B,E in Supplementary Material), thus revealing that the pro-survival effects exerted by BAFF on IgM^+^ cells are not due to negative effects on other populations. These results demonstrate that teleost BAFF, as mammalian BAFF (50), is exclusively a survival factor for IgM^+^ B cells.

### BAFF Increases IgM Production through Increased Survival of IgM^+^-Secreting Cells

**Figure 6:**
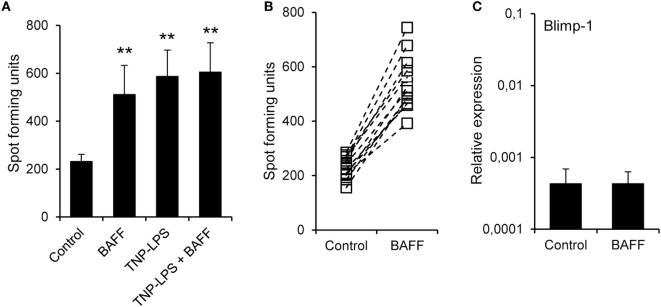
**B cell-activating factor (BAFF) increases the number of IgM-secreting cells**. **(A)** Splenocyte cultures were treated with BAFF (3 μg/ml), TNP-LPS (5 μg/ml), a combination of both, or left unstimulated (control) for 48 h and then plated in ELISPOT plates previously coated with anti-trout IgM mAb, for a further 24 h. After incubation, cells were washed away and a biotinylated anti-trout IgM mAb was used to detect numbers of spot forming cells. Quantification of spot forming cells is shown as mean + SD (*n* = 12, from four independent experiments containing three animals each). **(B)** The number of IgM-secreting cells was also plotted for each individual fish under control or BAFF stimulation conditions. **(C)** Splenocyte cultures were treated with BAFF (3 μg/ml) or left unstimulated for 24 h and, then, RNA from IgM^+^ FACS-isolated B cells was extracted as described in Section “Materials and Methods.” The transcription of Blimp-1 relative to the endogenous control EF-1α was calculated for each sample and shown as mean + SD (*n* = 12, from four independent experiments containing three animals each). Statistical differences were evaluated by a one-way ANOVA followed by a multiple comparison Tukey’s test, where **p ≤ 0.01.

**Figure 7:**
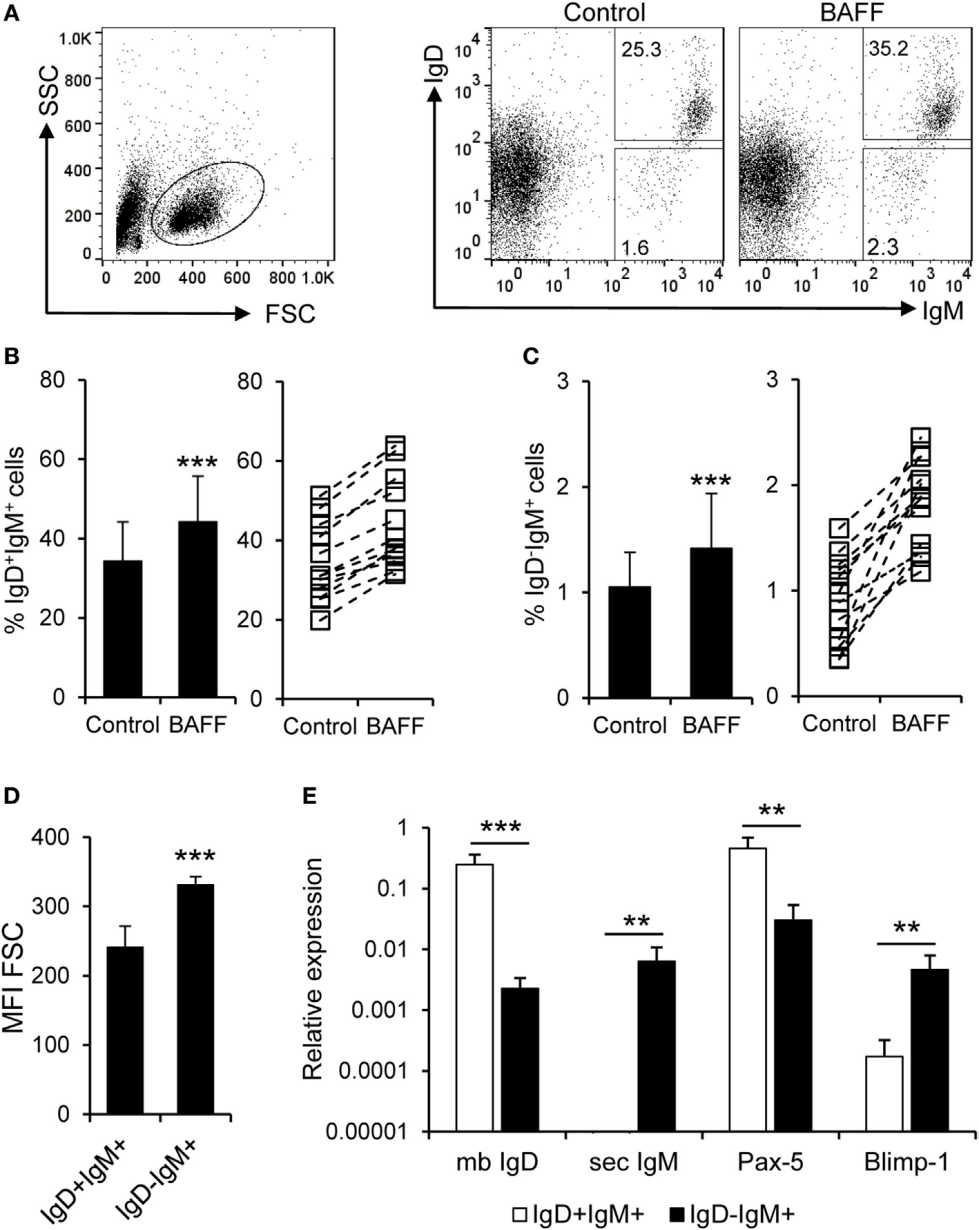
**B cell-activating factor (BAFF) promotes the survival of naïve B cells and IgM-secreting cells**. **(A)** Spleen leukocytes were incubated with recombinant BAFF (3 μg/ml) or left unstimulated (control) for 3 days at 20°C. After this time, cells were labeled with anti-IgD and anti-IgM mAbs and analyzed by flow cytometry. The percentage of live IgD^+^IgM^+^ and IgD^−^IgM^+^ B cells among the lymphocyte gate was then determined. Number of cells is annotated within the gates of the representative dot plots shown. Average survival of IgD^+^IgM^+^ **(B)** and IgD^−^IgM^+^ **(C)** B cell survival is shown (left panels) as well as the percentage of surviving B cells for each individual fish under control or BAFF stimulation conditions [**(B,C)**, right panels]. **(D)** IgD^+^IgM^+^ and IgD^−^IgM^+^ B cells were gated and the mean fluorescence intensity (MFI) for their forward scatter (FSC) determined (*n* = 12, from four independent experiments containing three animals each). **(E)** IgD^+^IgM^+^ and IgD^−^IgM^+^ B cells from the spleen were FACS isolated and RNA extracted. The transcription levels of membrane IgD (mb IgD), secreted IgM (sec IgM) Pax-5, and Blimp-1 relative to the endogenous control EF-1α were calculated for each sample and shown as mean + SD (*n* = 9, from three independent experiments containing three animals each). Dead cells were not excluded during the flow cytometry analysis, but cell viability was higher than 99% for IgM^+^ B cells and higher than 95% for other lymphocytes on these experimental settings (Figure S4C in Supplementary Material). Statistical differences were evaluated by a one-way ANOVA followed by a multiple comparison Tukey’s test, where **p ≤ 0.01 and ***p ≤ 0.005.

In mammals, BAFF has been shown to increase the secretion of IgM and IgG in the absence of immunization (51). Thus, we aimed to establish if recombinant trout BAFF could increase IgM secretion in splenocyte cultures. To carry this out, we incubated splenocytes for 48 h with BAFF and/or TNP-LPS, and after that time, we analyzed the number of IgM-secreting cells through ELISPOT. We observed a significant increase in the number of IgM-secreting cells after treatment with either BAFF or TNP-LPS, or a combination of both (Figure 6A). This increased number of IgM-secreting cells induced by BAFF was visualized in cell cultures from all fish examined (Figure 6B), but was not observed when a histidine-tagged irrelevant protein with a similar molecular weight was used (Figure S5C in Supplementary Material). Since Blimp-1 is an essential factor for the terminal differentiation of plasma cells in mammals (52), the fact that no upregulation of Blimp-1 expression was observed in sorted IgM^+^ B cells after treatment with BAFF (Figure 6C) suggested that IgM plasma cells were not differentiating from resting B cells in response to the cytokine. In mammals, BAFF has been shown to promote IgM secretion through increased survival of plasma cells (53), so it seemed plausible that the increase in the number of IgM-secreting cells in response to rainbow trout BAFF was a consequence of BAFF promoting the survival of a pre-existing IgM-secreting cell compartment (either plasmablasts or plasma cells) in the spleen. To test this hypothesis, we performed a double staining with anti-IgD and anti-IgM mAbs and analyzed the effect of BAFF on the survival of IgD^+^IgM^+^ cells and that of a small subpopulation of IgD^−^IgM^+^ B cells found in the spleen. We found that both IgD^+^IgM^+^ (Figures 7A,B) and IgD^−^IgM^+^ B cells (Figures 7A,C) had a significantly increased survival in the presence of BAFF, with consistent increases in all individual fish analyzed (Figures 7B,C). Because in mammals, activated B cells that start their differentiation toward plasmablasts/plasma cells lose IgD in the cell membrane (54), these two B cell subpopulations should correspond with naïve B cells (IgD^+^IgM^+^) and plasmablasts/plasma cells (IgD^−^IgM^+^). Confirming this premise, we phenotypically characterized these two B cell subpopulations. First, we estimated the size of these cells by means of the MFI of their forward scatter by flow cytometry, since an increase of the cell size is a common feature of antibody-secreting cells (ASCs) (55). As expected, IgD^−^IgM^+^ presented a significantly larger size than IgD^+^IgM^+^ B cells (Figure 7D). Furthermore, we, FACS isolated both B cell subtypes from the spleen and extracted RNA in order to study their gene expression pattern. Naïve IgD^+^IgM^+^ B cells had undetectable levels of secreted IgM and high-membrane IgD (mb IgD) and Pax5 transcription levels (Figure 7E). On the other hand, IgD^−^IgM^+^ cells presented significantly lower levels of mb IgD and Pax5 that, along with high mRNA levels of the secreted form of IgM, point to this population as an ASC type. Moreover, levels of Blimp-1 were significantly higher in IgD^−^IgM^+^ cells than in naïve B cells (Figure 7E). These results indicate that there is a tissue resident plasmablast/plasma cell population in the spleen of rainbow trout, and, as in mammals, IgM secretion is increased by BAFF through the increased survival of these IgM-secreting cells.

## Discussion

Fish are poikilotherm animals that regulate their metabolism in response to external temperature. Consequently, their immune system is also dependent on environmental conditions and even though teleost fish are the first animal group in which the complete adaptive immune machinery is present, at low temperatures, such as those at which salmonids usually live, these specific responses are significantly delayed. This, together with the lack of follicular structures in their immune organs, anticipates that, in teleost, the components of the adaptive immune system still retain many innate features and are closely dependent on innate regulatory factors. Given the fact that, in mammals, BAFF is a cytokine produced by innate cells upon antigen sensing, to regulate the survival and differentiation of B cells, especially at extrafollicular foci (56), we hypothesized that teleost B cell function will be tightly regulated by BAFF. Thus, in the current work, we have undertaken an extensive characterization of the effects of BAFF on the functionality of teleost B cells. Before undertaking these functional studies, we performed a transcriptional analysis to study BAFF mRNA levels throughout the different fish tissues in physiological conditions. We found that BAFF expression was highest in spleen, as reported in other fish species, with the exception of miiuy croaker, species in which highest BAFF mRNA levels were observed in skin (26). Consequently, our functional assays were performed with splenic IgM^+^ B cells.

In addition to providing an extensive characterization of B cell functions modulated by BAFF in teleost fish, the present study reports a major finding: the fact that, in teleost, a subset of B cells produces BAFF in physiological conditions. This result was confirmed through real-time PCR analysis, flow cytometry, and confocal imaging using a specific mouse polyclonal antibody against recombinant trout BAFF, previously characterized. Although only 7% of the IgM^+^ B cells produce BAFF as established by flow cytometry, given the high number of IgM^+^ B cells among splenocytes, this population accounts for approximately 28% of the cells producing BAFF pointing to an important contribution of this population on the homeostasis of the spleen. Of course, it would be possible that some of the B cells in our cultures have been previously exposed to an antigen as the fish used in our study were obtained from a fish farm. It must be noted that the BAFF-producing IgM^+^ B cells displayed a phenotype of resting mature B cells with high IgD expression on the cell membrane and transcription of surface IgM and IgD at high levels (data not shown), but we cannot discard that some of these cells may be antigen-experienced B cells, or even plasmablasts/plasma cells, given the fact that IgM-producing plasmablasts retain IgM on the cell membrane (57, 58). In mice and humans, BAFF is produced by macrophages, DCs, follicular DCs, stimulated neutrophils, and at low levels by T cells, but is never produced by resting B cells (3, 8, 59–62). Interestingly, B cells from B-cell chronic lymphocytic leukemia (63) or non-Hodgkin’s lymphoma (64), as well as B cells from patients with autoimmune disorders, namely rheumatoid arthritis (65), systemic lupus erythematosus (66), and primary Sjogren’s syndrome (67) also express BAFF, which rescues them from apoptosis in an autocrine loop. B1 cells and pre-B cells have also been shown to contain higher levels of BAFF mRNA when compared to transitional, follicular, or MZ B cells (68). Remarkably, all rabbit spleen B cells, known to express CD5 and consequently regarded as a B1-like population, also express BAFF constitutively (69). Thus, the fact that teleost B cells produce BAFF, adds up to previous evidences that point to the great similarity between teleost B cells and mammalian B1 populations. Interestingly, one of the distinctive features of B1 cells in all species, also shared by teleost B cells, is their extended survival, and this is a characteristic also seen in B cell disorders in which autocrine BAFF production has been reported (63–66). Therefore, BAFF seems to be a key factor involved in the prolonged survival of B1 cells, and this is something that should be further investigated.

Before addressing the effects that BAFF provoked on teleost B cell functions, we analyzed the binding of BAFF to splenocytes. Our results showed that only 8.4% of the IgM^+^ B cells in the spleen were able to bind to recombinant BAFF; however, this percentage went up to 39.2% when both binding and internalization were determined through confocal microscopy. On the other hand, our experiments also revealed that 3.9% of the IgM^−^ cells in the rainbow trout spleen also bound recombinant BAFF. Taking into account that IgT^+^ B cells in the rainbow trout spleen constitute 25% of the total B cell population, it seems plausible that these IgM^−^ lymphocytes binding BAFF are in fact IgT^+^ B cells and this is something that should be further explored. The percentage of B cells binding BAFF in the spleen revealed by our experiments seemed surprisingly low when compared to mammals as all splenic human (53) and mouse B cells (70) bind BAFF through BAFF-R, constitutively expressed on the cell surface of all resting B cells. We have very recently demonstrated that rainbow trout splenic IgM^+^ B cells transcribe not only BAFF-R but also BCMA (unpublished observations), thus it does not seem probable that this lower capacity to bind exogenous BAFF is a consequence of lower receptor expression levels. Another possible explanation is that BAFF receptors in trout B cells are already occupied by endogenous BAFF produced constitutively in the spleen. In rabbit, where B cells have been shown to actively produce BAFF, a low BAFF-binding capacity of splenic B cells has also been shown (69). The authors similarly concluded that these cells could not bind recombinant BAFF because the receptors were already occupied with endogenously synthesized BAFF.

B cells are professional antigen-presenting cells (APCs), which can endocytose antigens through the BCR and effectively present antigens to T cells in an MHC II context (71). In teleost, the high phagocytic capacity of B cells predicts an even higher antigen-presenting capacity than that of mammalian B cells (37), also being capable of presenting particulate antigens. Thus, initially, we studied the effect of BAFF on both the surface MHC II expression levels and the phagocytic capacities of splenic IgM^+^ B cells. We found that BAFF strongly increased surface MHC II expression in IgM^+^ cells, at levels much higher than those reached by TNP-LPS-treated IgM^+^ B cells. Because MHC II gene transcription was not upregulated in BAFF-treated cells, it appears that this increase is a consequence of increased translocation of MHC II molecules to the plasma membrane or an augmented half-life of membrane MHC II. A similar increase in surface MHC II expression in B cells in response to BAFF has also been demonstrated in mammals (18, 72). Although this increased surface MHC II expression anticipates an increased antigen-presenting capacity of B cells, the uptake of particulate antigen through phagocytosis was not affected by BAFF, indicating that BAFF only regulates the posterior antigen exposure on the membrane.

Focusing further on the effects that BAFF produces on teleost B cells, we observed insignificant proliferative effects, in contrast to the high proliferative effects exerted by TNP-LPS. Despite this, there was a significant increase in IgM^+^ B cell survival, at levels similar to those of TNP-LPS, demonstrating that in the absence of additional stimulation, rainbow trout BAFF is exclusively a survival factor for IgM^+^ B cells, as occurs in mammals (11, 50). This extended survival was observed in naïve B cell populations of the spleen that co-expressed IgM and IgD in the cell membrane as well as in plasmablasts that have lost surface IgD once the differentiation process toward plasmablast/plasma cell has begun. This plasmablast/plasma cell phenotype was confirmed by the enlarged size of these IgD^−^IgM^+^ cells, together with the expression of the secreted form of IgM and the increased transcriptional levels of Blimp-1, thus indicating that BAFF can act in different splenic trout B cell subsets, as it happens in mammals (73). This is the first characterization of such B cell subpopulations in teleost by means of surface IgD expression. The extended survival of these plasmablasts/plasma cells is the most probable cause for the increase in the number of IgM-secreting cells observed after BAFF treatment of splenocyte cultures. In mammals, there is some controversy regarding the effects of BAFF on antibody secretion. In mice, the administration of BAFF alone, increased serum IgM and IgA levels, but had no effect on IgG (59), again pointing to preferred BAFF actions outside the GC. On the other hand, transgenic mice overexpressing BAFF had increased IgG levels, while IgM levels were only slightly augmented (72). Finally, when BAFF was administered in combination with a *Streptococcus pneumoniae* vaccine, IgM, and IgG were only marginally affected, whereas BAFF markedly increased IgA production (51). In trout, we have established that BAFF increases the secretion of IgM in the spleen in a Blimp-1-independent fashion, ruling out a role of BAFF as a B cell differentiation factor. This increased IgM production seems to be a direct consequence of the increased survival of pre-existing IgM-secreting cells.

In conclusion, we have demonstrated that different teleost B cell populations respond to BAFF. Splenic IgM^+^ B cells, the main cell type producing BAFF in this tissue, are able to extend their survival and upregulate their MHC II surface levels in response to the cytokine. Splenic plasmablasts do not produce BAFF in physiological conditions (data not shown) but also have an increased survival rate in response to the cytokine, which results in increased IgM secretion levels. Our results reveal an ancient mechanism through which BAFF regulates B cell function in teleost. To our knowledge, this is the first study that demonstrates this unique regulatory pathway on B cells under physiological conditions. Thus, our findings unravel the ancient origin of this regulatory mechanism that reemerges in a variety of B cell disorders such as autoimmune diseases or B cell malignancies.

## Author Contributions

CT performed data analysis and collaborated in the design of the experiments. LG carried out RNA extractions, cDNA synthesis, and real-time PCRs. LG also assisted RC and AG in tissue sampling and processing. RC performed the cell proliferation and survival experiments. AG performed the rest of the experimental work, performed data analysis, and designed the experiments together with CT. CT and AG wrote the paper.

## Conflict of Interest Statement

The authors declare that the research was conducted in the absence of any commercial or financial relationships that could be construed as a potential conflict of interest.
