# Supplementary material for: B Cell-Activating Factor Regulates Different Aspects of B Cell Functionality and Is Produced by a Subset of Splenic B Cells in Teleost Fish
Source: Front Immunol. 2017 Mar 15;8:295. doi: 10.3389/fimmu.2017.00295 (PMC5350146; doi:10.3389/fimmu.2017.00295)
Supplement: Supplementary file 1 [file Data_Sheet_1.PDF]

Supplemental Figure 1

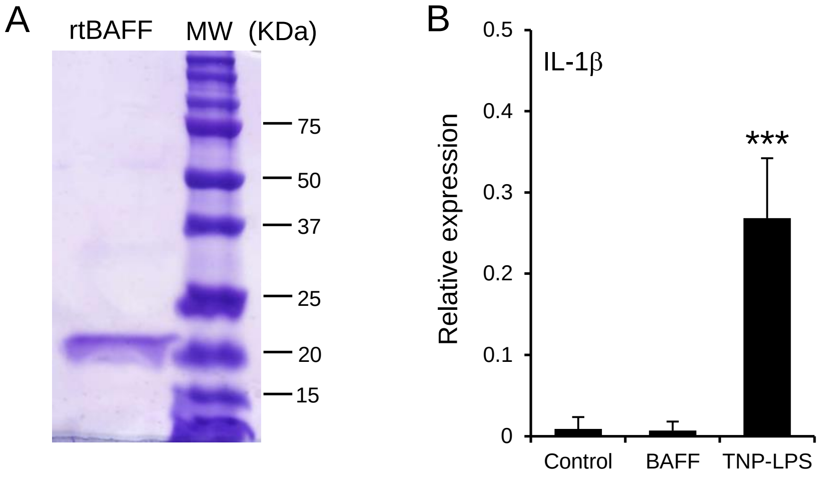

Supplemental Figure 1. Production of recombinant rainbow trout BAFF. (**A**) Rainbow trout recombinant BAFF protein (2.5 μg) was loaded on a 12% polyacrylamide gel and SDS-PAGE was performed. The gel was then stained with Coomasie reagent to show the purity of the recombinant protein. (**B**) Rainbow trout splenocyte cultures were treated with BAFF (3 μg/ml), TNP-LPS (5 μg/ml), or left unstimulated (control) for 24 h, and RNA was then extracted as described in Materials and Methods. The expression of IL-1β gene relative to the endogenous control gene EF-1α was calculated for each sample, and shown as mean + SD (n=12). Statistical differences were evaluated by one-way ANOVA followed by a multiple comparison Tukey´s test , where *** means $p \leq 0.005$.

Supplemental Figure 2

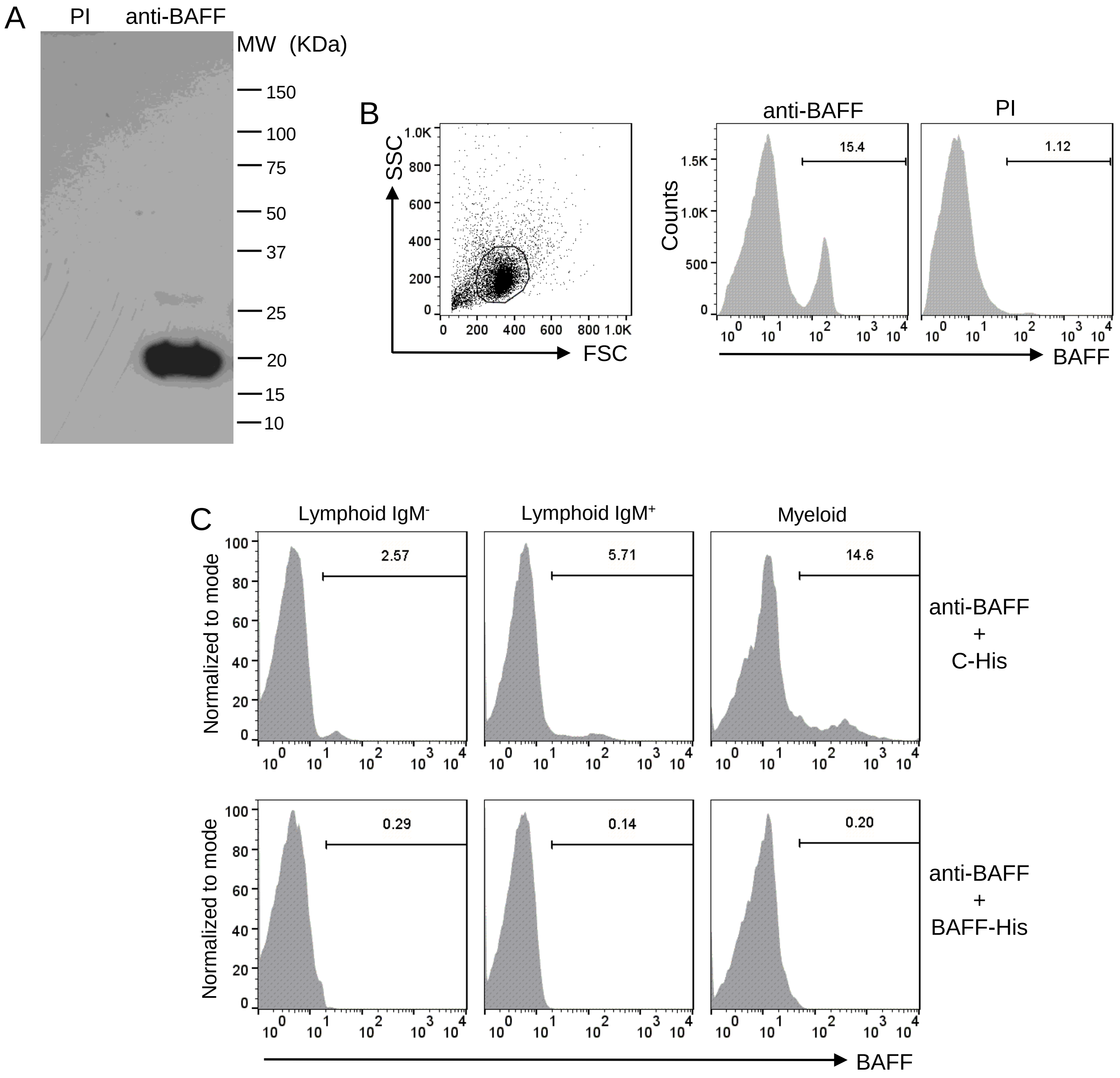

Supplemental Figure 2. Characterization of the mouse pAb generated against rainbow trout BAFF. (**A**) To analyze the specificity of the anti-BAFF pAb, recombinant rainbow trout BAFF (10 ng per lane) was used to test the anti-BAFF pAb by Western blot as described in Methods. Serum from immunized mice (anti-BAFF) was compared to pre-immunized serum (PI) from the same animal. The BAFF pAb recognizes the recombinant protein (extracellular domain of BAFF) at the expected molecular weight (20.7 Kda). (**B**) Total leukocytes from spleen were incubated with anti-BAFF or a preimmune (PI) serum, then stained with an anti-mouse IgG-Alexa Fluor 488, and analyzed by flow cytometry. FSC/SSC profile is shown (left), lymphocytes were gated and BAFF staining on the lymphocyte gate is shown as histograms (right). (**C**) Rainbow trout leukocytes isolated from spleen were stained with an anti-IgM mAb together with an anti-BAFF pAb, which had been previously pre-incubated with C-His (upper row) of BAFF-His (lower row) proteins for 1 h (molar ratio 1:10). Samples were analyzed by flow cytometry and IgM$^-$ and IgM$^+$ were selected from the lymphoid gate, as well as total cells from the myeloid gate, following the same strategy described in Figure 2. A representative image from five independent experiments is shown.

Supplemental Figure 3

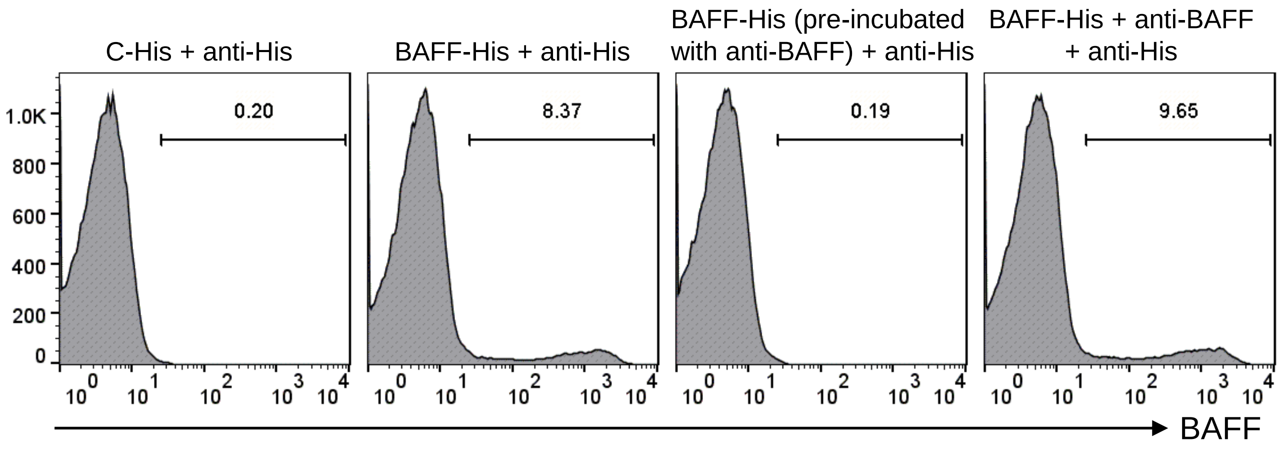

Supplemental Figure 3. Pre-incubation of BAFF with anti-BAFF pAb specifically blocks the binding of BAFF to IgM+ B cells. To analyze whether the anti-BAFF pAb was specifically blocking the binding of BAFF to the cells, freshly isolated splenic leukocytes were incubated with recombinant histidine-tagged BAFF protein (3 μg/ml) or an irrelevant 20 KDa histidine-tagged protein (C-His, 3 μg/ml) for 1 h. In parallel, cells were also cultured for 1 h with recombinant BAFF protein which had been previously incubated for 1 h with an anti-BAFF pAb (molar ratio 1:10). Cells cultured in the presence of histidine-tagged BAFF protein for 1 h were then incubated for an additional hour with an anti-BAFF pAb (molar ratio 1:10). Thereafter, all samples were stained with an anti-IgM mAb together with an anti-His mAb, or with isotype control mAbs, and analyzed by flow cytometry. Histograms from one representative experiment are shown (n=6). Percentages of BAFF$^+$ cells within the IgM$^+$ lymphoid gate are shown for each histogram.

Supplemental Figure 4

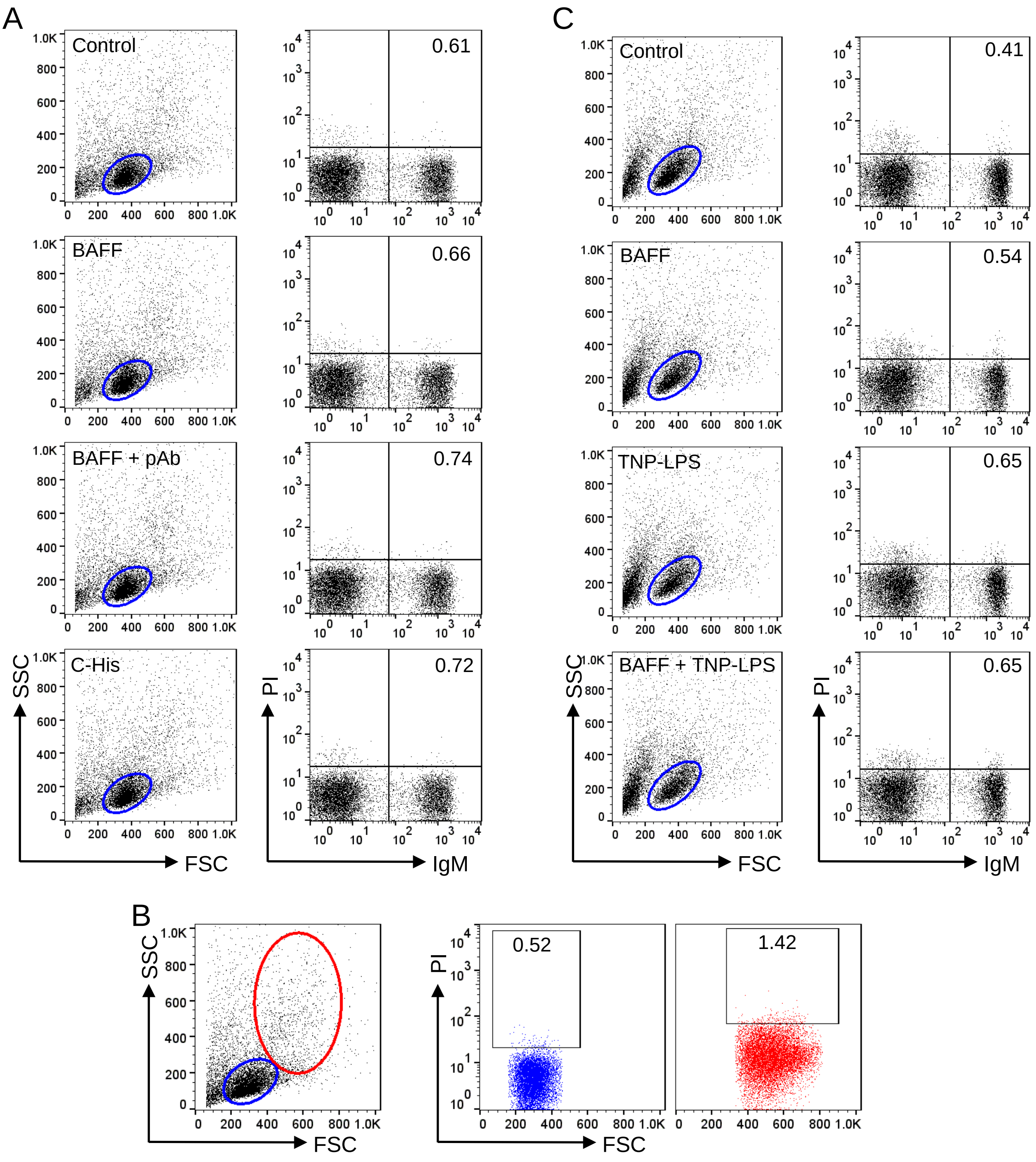

Supplemental Figure 4. Cell viability on rainbow trout splenocytes. (**A**) Freshly isolated splenic leukocytes were incubated with recombinant histidine-tagged BAFF protein (3 µg/ml) or an irrelevant 20 KDa histidine-tagged protein (C-His, 3 µg/ml) for 1 h. In parallel, cells were also cultured for 1 h with recombinant BAFF protein which had been previously incubated for 1 h with an anti-BAFF pAb (molar ratio 1:10). Thereafter, cells were stained with an anti-IgM mAb for 30 min, and then incubated with 10 µg/ml Propidium Iodide (PI) for 5 min, and analyzed by flow cytometry. (**B**) Rainbow trout leukocytes isolated from spleen were analyzed by flow cytometry. Lymphoid (blue gate) and myeloid (red gate) populations were gated, and cell viability was tested by PI staining. (**C**) Spleen leukocytes were incubated with recombinant BAFF (3 µg/ml), TNP-LPS (5 µg/ml), a combination of both, or left unstimulated (control) for 3 days at 20°C. After this time, cells were labeled with an anti-IgM mAb, and cell viability was analyzed by flow cytometry by means of PI staining. In all cases, a representative example of three independent experiments is shown. Numbers inside the plots represent the percentage of dead cells within the IgM+ population (**A**, **C**) and the percentage of dead cells within the lymphoid and myeloid gates (**B**).

Supplemental Figure 5

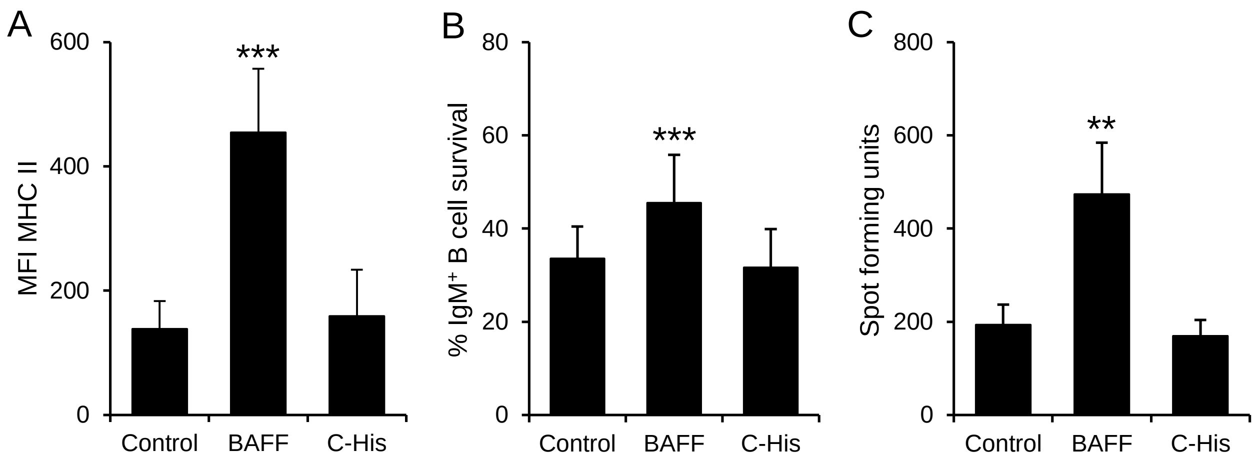

Supplemental Figure 5. Specificity of the effects triggered by BAFF on spleen B cells. To analyze the specific activity of BAFF on IgM$^+$ B cells and rule out potential effects of the histidine tag fused to the protein, several parameters were tested. Freshly isolated splenic leukocytes were incubated with recombinant histidine-tagged BAFF protein (3 μg/ml), with an irrelevant 20 KDa histidine-tagged protein, (C-His, 3 μg/ml), or left unstimulated (Control). After 3 days of incubation at 20°C, cells were labeled with anti-IgM and anti-MHC II mAbs and analyzed by flow cytometry. (**A**) The mean fluorescence intensity (MFI) of MHC II on IgM$^+$ B cells was then determined. Average MFI is shown as mean + SD. (**B**) The percentage of live IgM$^+$ B cells among the lymphocyte gate was also determined. Quantification of average B cell survival is shown as mean + SD. (**C**) Splenic leukocytes incubated in the same conditions for 2 days at 20°C were plated in ELISPOT plates previously coated with anti-trout IgM mAb, for a further 24h. After incubation, cells were washed away and a biotinylated anti-trout IgM mAb was used to detect numbers of spot forming cells. Quantification of spot forming units is shown as mean + SD. (n=9, from three independent experiments containing three animals each). Statistical differences were evaluated by one-way ANOVA followed by a multiple comparison Tukey´s test, where ** means $p \leq 0.01$ and *** means $p \leq 0.005$.

Supplemental Figure 6

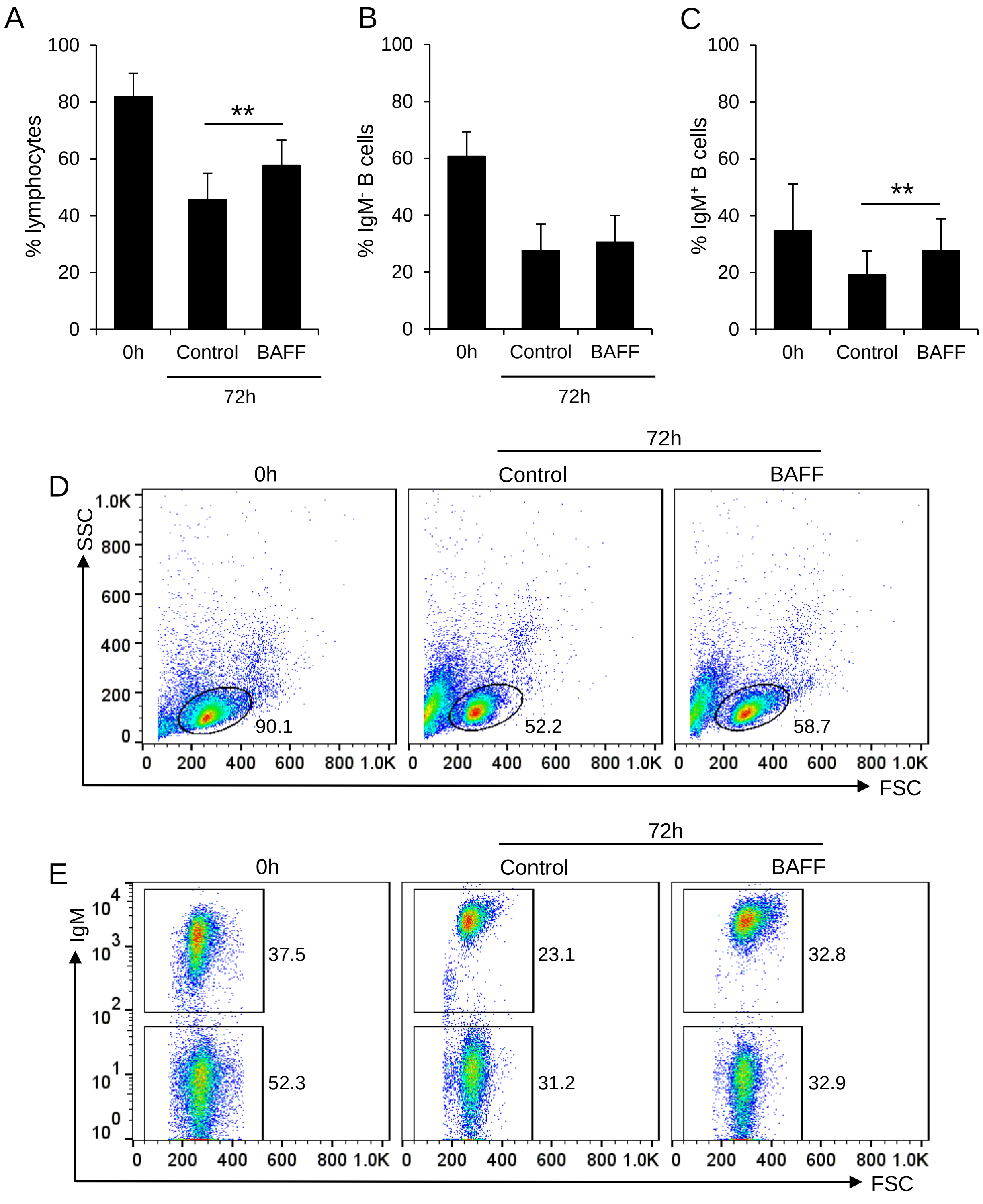

Supplemental Figure 6. Specificity of the effects triggered by BAFF on spleen IgM- and IgM+ lymphocytes. Spleen leukocytes were incubated with recombinant BAFF (3 µg/ml) or left unstimulated (control) for 72 h at 20°C. At times 0 h and 72 h post-incubation, cells were labeled with an anti-IgM mAb and analyzed by flow cytometry. The percentage of lymphocytes within total splenocytes was then determined (**A**). In parallel, the percentages of IgM^- (**B**) and IgM^+ (**C**) cells among total live cells in the cultures were also determined. Results are shown as mean + SD. (n=9, from three independent experiments containing three animals each). A representative dot plot is shown for the quantification of lymphocytes (**D**) and the quantification of IgM^- and IgM^+ cells (**E**). Statistical differences were evaluated by one-way ANOVA followed by a multiple comparison Tukey´s test, where ** means p ≤ 0.01.

Supplemental Table 1

Gene	Forward primer (5’-3’)	Reverse primer (5’-3’)
BAFF	ATGTTTGATGCTTATTCTGGCAGGT	TGGGACTGTGTCTTGACTGTGTGTA
Blimp-1	GGCAGTGGACCTGTGGAAGG	CGCAGGTGGACCTTGAGGTT
EF1α	GATCCAGAAGGAGGTCACCA	TTACGTTCGACCTTCCATCC
mb IgD	CAGGAGGAAAGTTCGGCATCA	CCTCAAGGAGCTCTGGTTTGGA
sec IgM	CCTTAACCAGCCGAAAGGG	TGAGGTTCTATCAATGGTTCTC
IL-1β	CTGAAGCCAGACCTGTAGCC	GCAACCTCCTCTAGGTGCAG
MHC-II	ACACCCTTATCTGCCACGTC	TCTGGGGTGAAGCTCAGACT
Pax-5	ACGGAGATCGGATGTTCCTCTG	GATGCCGCGCTGTAGTAGTAC

Supplemental Table 1. Real time PCR Primers used in this study. Gene names and forward and reverse primer sequences are indicated.
